# Mid-Infrared Photonic Sensors: Exploring Fundamentals, Advanced Materials, and Cutting-Edge Applications

**DOI:** 10.3390/s25041102

**Published:** 2025-02-12

**Authors:** Muhammad A. Butt, Marcin Juchniewicz, Mateusz Słowikowski, Łukasz Kozłowski, Ryszard Piramidowicz

**Affiliations:** 1 Warsaw University of Technology, Institute of Microelectronics and Optoelectronics, Koszykowa 75, 00-662 Warsaw, Poland; 2 Warsaw University of Technology, The Centre for Advanced Materials and Technologies CEZAMAT, Poleczki 19, 02-822 Warsaw, Poland

**Keywords:** mid-infrared, molecular fingerprint, sensors, environmental monitoring, biomedical, chemical and gas sensing

## Abstract

Mid-infrared (MIR) photonic sensors are revolutionizing optical sensing by enabling precise chemical and biological detection through the interrogation of molecules’ unique vibrational modes. This review explores the core principles of MIR photonics, emphasizing the light–matter interactions within the 2–20 µm wavelength range. Additionally, it examines innovative sensor architectures, such as integrated photonic platforms and optical fibers, that enhance sensitivity, specificity, and device miniaturization. The discussion extends to groundbreaking applications in environmental monitoring, medical diagnostics, industrial processes, and security, highlighting the transformative impact of these technologies. This comprehensive overview aims to illuminate the current state-of-the-art while inspiring future developments in MIR photonic sensing.

## 1. Introduction

Mid-infrared (MIR) photonic sensors, operating in the 2–20 μm wavelength range, are rapidly gaining importance due to their exceptional capabilities for molecular sensing [1,2]. This part of the spectrum, known as the “molecular fingerprint” region, corresponds to the fundamental vibrational and rotational energy levels of many molecules, allowing for highly selective and sensitive detection. Unlike other spectral regions, the MIR range enables direct access to these fundamental absorption bands, making it possible to detect small concentrations of various substances with high specificity and minimal interference. As a result, MIR photonic sensors offer distinct advantages over traditional detection methods, which often rely on indirect or less sensitive approaches. Moreover, these sensors can provide real-time, non-invasive, and in situ analysis, a crucial feature for applications requiring rapid and reliable information [3]. 

Quantum cascade lasers (QCLs) [4] and interband cascade lasers (ICLs) [5] are two key MIR light sources with distinct operating principles and application domains. QCLs rely on intersubband transitions within a single conduction band, enabling operation at a wide range of MIR wavelengths (typically 3–25 µm) with high output power and broadband tunability. They are particularly suited for high-power applications such as healthcare, remote sensing, free-space communication, and environmental gas detection [6,7]. In contrast, ICLs utilize interband transitions between the conduction and valence bands, making them highly efficient at lower MIR wavelengths (typically 3–6 µm) with lower threshold currents and reduced power consumption. Due to their higher wall-plug efficiency and compact size, ICLs are well suited for portable gas sensing, medical diagnostics, and industrial process monitoring [8]. While QCLs dominate high-power applications, ICLs offer an energy-efficient alternative for battery-operated and field-deployable sensing systems, making both technologies complementary for various MIR photonic applications [9]. Today, these sensors are a critical component of modern sensing technologies, offering unprecedented molecular detection capabilities across multiple domains [1,10,11].

Integrated photonic platforms leverage compact, chip-based technologies to create components like waveguides, resonators, interferometers, and detectors tailored to the MIR range [12,13,14]. Materials such as silicon, silicon nitride, germanium, and chalcogenide glasses are commonly used due to their transparency and performance in this wavelength range. Devices such as photonic crystal cavities [15,16], micro-ring resonators [17,18], and Mach–Zehnder interferometers [19] can be engineered to enhance sensitivity and selectivity, improving the detection limits of trace gases or pollutants [20,21]. With advances in on-chip light sources and detectors, these integrated photonic devices offer a scalable, cost-effective alternative to traditional bulkier MIR systems, making MIR sensing more accessible and versatile across various fields [22,23,24,25].

One of the primary applications of MIR photonic sensors is in environmental monitoring, where they are invaluable for detecting greenhouse gases, pollutants, and other hazardous substances in real-time [26,27,28]. MIR sensors provide accurate measurements of gases like carbon dioxide (CO_2_), methane (CH_4_), and nitrous oxide (N_2_O), which exhibit strong absorption bands within the MIR range, aiding in the monitoring and management of air quality [29]. Additionally, MIR photonic sensors are used to assess water quality by detecting organic pollutants and toxic compounds, contributing significantly to environmental protection initiatives. With their high sensitivity and ability to provide real-time data, these sensors support ongoing efforts to address environmental challenges effectively. In the field of biomedical diagnostics, MIR photonic sensors are advancing non-invasive diagnostic techniques through applications in breath analysis, tissue imaging, and biofluid analysis. Human breath, for instance, contains biomarkers that serve as indicators of various diseases, ranging from respiratory conditions to metabolic disorders. By analyzing these molecular fingerprints, MIR sensors can help diagnose these conditions early and accurately. They are also being explored for cancer marker detection and blood glucose monitoring, paving the way for more personalized and preventive healthcare solutions [30]. The ability of MIR sensors to offer real-time analysis without requiring invasive procedures makes them an attractive choice in modern medical diagnostics [31,32].

**Figure 1:**
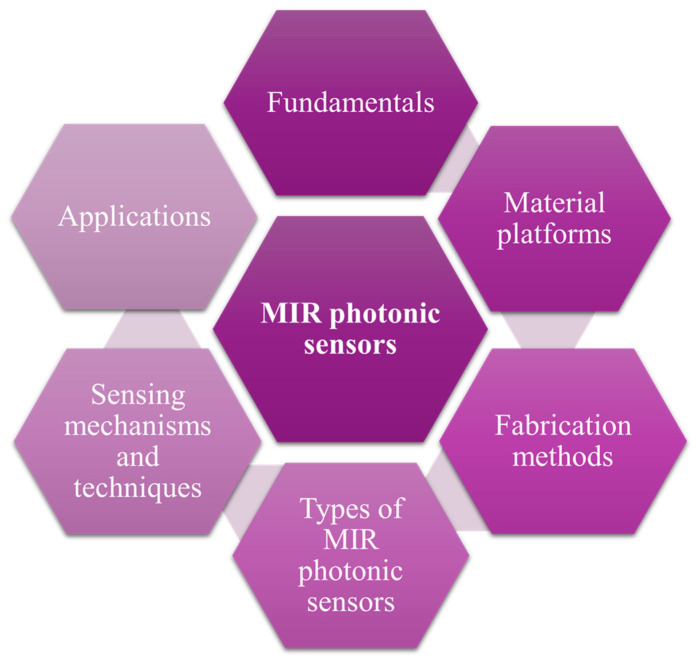
A structured outline illustrates the paper’s content’s logical flow and progression.

MIR photonic sensors are also crucial in industrial process control, particularly in the monitoring of chemical processes, combustion systems, and emission control. In manufacturing environments, real-time sensing allows operators to optimize processes, detect faults at early stages, and ensure compliance with environmental regulations. By monitoring specific molecular signatures, MIR sensors improve process efficiency and product quality while minimizing waste and energy consumption. In industries such as petrochemicals and pharmaceuticals, where precise control over chemical composition is essential, MIR photonic sensors provide reliable and accurate measurements, enhancing both safety and productivity [1,33,34]. In defense and security applications, MIR photonic sensors play a vital role in the detection of explosives, toxic chemicals, and other hazardous materials. Their ability to identify substances based on their unique molecular signatures allows for rapid screening of potentially dangerous compounds in public areas, military settings, and border security checkpoints. Furthermore, MIR sensors contribute to infrared countermeasures and thermal imaging applications, improving the ability to detect and identify threats in challenging environments. By providing rapid, reliable, and non-invasive detection capabilities, MIR photonic sensors significantly enhance security and defense measures [35,36]. The combination of molecular specificity, sensitivity, and adaptability makes MIR photonic sensors indispensable across these diverse fields [37]. As MIR technology continues to evolve, its application in the environmental, biomedical, industrial, and security sectors is expected to grow, contributing to a safer, healthier, and more sustainable world [38,39,40]. Figure 1 presents a structured outline of the paper’s content’s logical flow and progression.

## 2. Fundamentals, Material Platforms, and Fabrication Methods of MIR Photonics

In this section, we have provided a concise yet comprehensive overview of the core principles of MIR photonics, explored various material platforms, and detailed the fabrication techniques employed in developing MIR photonic devices.

### 2.1. Fundamentals of MIR Photonics

The MIR region is particularly significant in molecular sensing due to its ability to probe fundamental vibrational transitions in molecular bonds. Molecular vibrations, such as stretching, bending, and torsional modes, interact with MIR photons, resulting in distinct absorption features that are characteristic of specific chemical bonds, including C-H, O-H, N-H, C=O, and C≡N [41,42,43]. In the MIR region, molecules exhibit unique absorption spectra due to complex vibrational combinations and overtones that enable high specificity in molecular identification. This range is advantageous for molecular sensing because it enables vibrational spectroscopy [44], allowing for the detailed characterization of molecular structures through specific bond interactions. Each molecular bond has a unique vibrational frequency within the MIR range, meaning MIR spectroscopy can effectively identify individual molecules and complex mixtures based on their unique spectral profiles. This capability is essential for applications requiring molecular discrimination and quantification, such as chemical analysis, environmental monitoring, and biomedical diagnostics [45]. Furthermore, the MIR spectral region enables label-free, non-destructive analysis, eliminating the need for additional reagents or sample modifications, which preserves sample integrity. Additionally, the MIR region is highly effective for detecting trace-level concentrations of gases, liquids, and solids due to the strong, specific absorption coefficients of molecular bonds in this range. The MIR’s sensitivity to concentration allows quantitative analysis, commonly utilizing the Beer–Lambert law, which correlates absorbance to the analyte concentration in a sample [26,46,47]. 

MIR photonic sensors operate based on interactions between MIR light and molecular structures within a sample, leveraging mechanisms such as absorption, transmission, and reflection. These interactions provide valuable data on the sample’s molecular composition, concentration, and, in some cases, its physical state. The sensing mechanism is fundamentally based on the molecular vibrational modes that occur when molecules absorb MIR photons, resulting in measurable spectral signatures. These signatures can reveal detailed information about the sample’s molecular composition.

(I) Absorption: In MIR sensing, absorption is the primary mechanism of interaction. When MIR light passes through or interacts with a sample, molecules absorb specific wavelengths corresponding to their vibrational frequencies, creating excitation of bonds within the molecule [48]. The wavelengths at which this absorption occurs are unique to specific molecular bonds and functional groups, resulting in a characteristic spectrum that serves as a “molecular fingerprint.” By analyzing the absorption spectrum, it is possible to identify and quantify various compounds within a mixture. This is especially useful in applications requiring precise molecular identification, as each molecular bond and functional group has a distinct spectral location within the MIR region [49].
(II) Transmission: Transmission-based MIR sensing involves passing MIR light through a sample and measuring the transmitted light. As the light travels through the sample, certain wavelengths are selectively absorbed by specific molecular bonds [50]. The remaining transmitted light intensity, recorded as a function of wavelength, reveals both qualitative and quantitative information about the sample’s composition. Transmission spectroscopy is particularly effective for gas-phase or thin-film samples, where minimal sample thickness allows for clear and interpretable spectra [51]. The Beer–Lambert law is frequently applied in transmission-based MIR sensing to calculate the concentration of analytes by relating the measured absorbance to the analyte concentration within the optical path [52].
(III) Reflection: Reflection-based MIR sensing is commonly employed for samples that are opaque or highly absorbing, where transmission is impractical. In this approach, MIR light is directed onto the sample surface, and the reflected light is measured [53]. One of the most widely used reflection techniques is attenuated total reflectance (ATR) spectroscopy, which involves coupling MIR light into a high-refractive-index crystal [54]. The light undergoes multiple total internal reflections within the crystal, creating an evanescent wave that extends slightly into the sample in contact with the crystal. This wave interacts with the sample, resulting in absorption at specific MIR wavelengths. ATR spectroscopy is highly versatile and enables the analysis of a wide range of media, including solids, liquids, and gels, by using various crystal materials to optimize light coupling with different sample types [55].
(IV) Frequency upconversion: Frequency upconversion is a powerful technique that enhances the detection and resolution of signals in the MIR region [56,57]. By converting MIR signals to higher-frequency signals, such as NIR or visible light, this method enables the use of more efficient and sensitive detectors that are commonly available [58,59]. The upconversion process, often achieved through nonlinear optical interactions or multi-photon absorption, improves the signal-to-noise ratio and allows for enhanced imaging and sensing capabilities [60]. This is particularly valuable in applications like chemical sensing, gas detection, and biomedical imaging [61], where MIR wavelengths are crucial for molecular vibration analysis, but their direct detection can suffer from noise or sensitivity limitations [62]. By upconverting these MIR signals to more detectable wavelengths, the technique provides clearer, more accurate measurements and opens up new possibilities for real-time, high-resolution imaging and sensing in fields such as environmental monitoring, medical diagnostics, and industrial inspections [63,64].

### 2.2. Material Platforms

In this section, several known material platforms for MIR photonics are discussed. Moreover, Table 1 summarizes the widely used material platforms for MIR photonics. 

#### 2.2.1. Silicon-Based Photonics

Silicon-on-insulator (SOI) photonic platforms are among the most extensively utilized technologies in integrated photonics. SOI substrates are predominantly procured from commercial suppliers, reflecting their standardized and widely available nature [65,66,67,68]. A multitude of organizations provide commercial multi-project wafer (MPW) services tailored for SOI-based photonics, facilitating the fabrication of both passive components [69,70], such as waveguides, couplers, gratings, resonators, and active devices, including integrated photodiodes and thermo-optical and optical modulators. This inherent versatility positions SOI-based photonic integrated circuits (PICs) as an ideal choice for a broad spectrum of applications, encompassing telecommunications, data communications across C, L, and O bands, optical sensing, and LiDAR systems.

**Figure 2:**
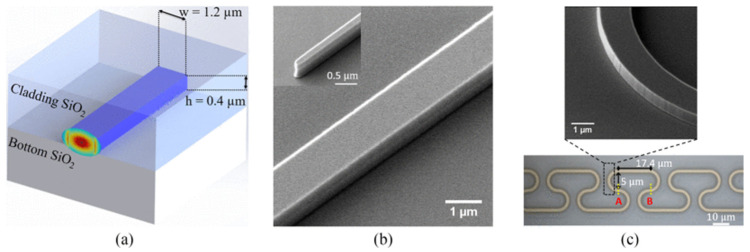
An example of an MIR waveguide on an SOI platform demonstrates propagation losses ranging from 2 to 3 dB/cm across the broad MIR spectrum of 3.68–3.88 μm. The bending losses were as low as 0.02 dB per 90° turn for radii larger than 10 μm. The figure includes (**a**) a schematic of the single-mode SOI channel waveguide, (**b**) SEM image of the straight waveguide with an inset of the inverse taper tip, and (**c**) optical images of waveguide bends with a radius of 5 μm [65].

SOI-based PICs are predominantly designed with silicon structures positioned on a buried oxide (BOX) layer (Figure 2). This architecture forms the foundation of the widely used commercial platforms. Typically, photonic components are fabricated via dry etching, primarily utilizing fluorine-based plasmas, though the specifics can vary. For example, reactive ion etching (RIE) with CHF_3_/O_2_/SF_6_ has been employed to create rib waveguides, achieving propagation losses of 3.4 ± 0.2 dB/cm at 3.8 µm and 2.9 ± 0.3 dB/cm at 3.73 µm [71]. Deep Reactive Ion Etching (DRIE) surpasses conventional RIE in applications requiring high-aspect-ratio structures, such as optical waveguides. DRIE achieves significantly higher ratios, often exceeding 10:1, while RIE is typically limited to lower ratios. It also produces nearly vertical sidewalls, which are crucial for effective optical mode confinement, and offers faster etch rates, making it ideal for deep etching [72]. 

Additionally, DRIE, when properly optimized, causes less surface damage compared to RIE, which can introduce defects due to continuous ion bombardment. However, the Bosch process in DRIE can create scalloped sidewalls, leading to increased scattering losses in waveguides. This issue can be mitigated through post-processing techniques like isotropic etching or thermal oxidation, which smooth the sidewalls and reduce propagation loss. When carefully optimized, DRIE provides superior waveguide performance by ensuring better mode confinement and minimizing optical losses. DRIE with SF_6_/C_4_F_8_, in combination with e-beam lithography, has been used to fabricate MMIs and waveguides with propagation losses as low as 1.00 ± 0.08 dB/cm [73].

Standard UV lithography followed by SF_6_/O_2_/CHF_3_ plasma has been utilized for rib waveguide production, whereas alternating plasma cycles of SF_6_/O_2_ (etch) and C_4_F_8_ (passivation) were applied to fabricate strip waveguides [71]. Reported propagation losses for strip waveguides were 4.9 ± 0.2 dB/cm (W = 1.2 µm), 4.5 ± 0.3 dB/cm (W = 1.4 µm), and 4.2 ± 0.2 dB/cm (W = 1.6 µm) at a wavelength of 3.39 µm. Rib waveguides showed propagation losses of 3.4 ± 0.2 dB/cm at 3.8 µm and 2.9 ± 0.3 dB/cm at 3.73 µm.

The spectral range of SOI devices is restricted to 3.5 µm due to silica’s absorption characteristics. To address this limitation, techniques such as suspending waveguides are employed [74]. This can be achieved using wet etching in HF solutions. For instance, one study utilized an SOI wafer (device layer: 340 nm, BOX layer: 340 nm) with silicon layer openings (hole radius: ~200 nm, spacing: 530 nm). The wafer underwent etching in 49% HF for 30 s, followed by rinsing in deionized water [75]. Another structure with a 220 nm device layer, a 1000 nm BOX layer, and holes of 240 nm radius (spaced 430 nm apart) was suspended using a diluted HF solution (1:10 HF: water), although the etching duration was unspecified [76]. Notably, gaps smaller than 150 nm in cladding were unsuitable for liquid etching [76]. For narrower openings, such as subwavelength grating (SWG) cladding in waveguides, HF vapor etching was required. However, this method extended the process and introduced complexities due to water accumulation as a reaction by-product, causing the etching rate to drop from 30 nm/min to nearly zero within 3–5 min. Sample dehydration at 220 °C was necessary before continuing. Ultimately, a 450 nm gap was achieved, enabling etching in an HF solution (1:7 HF: water) for 30 min, which produced an etch depth of 2.5 µm. Waveguide propagation loss improved from 5 dB/cm at 2.75 µm to 3.6 dB/cm after etching.

A key advantage of silicon-on-sapphire (SOS) devices over SOI devices is the absence of silica, which strongly absorbs beyond a 3.6 µm wavelength [77]. This limitation in SOI devices often necessitates measures like waveguide suspension. By contrast, sapphire substrates in SOS devices exhibit low absorption up to 5–6 µm and possess a relatively low refractive index compared to silicon [78]. However, operating at wavelengths beyond this range is challenging, effectively capping the spectral utility of SOS platforms at approximately 6 µm.

**Figure 3:**
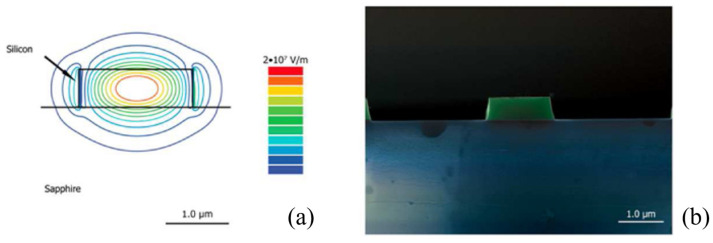
(**a**) A contour plot of the optical mode of the SOS waveguide, (**b**) a false-color scanning electron micrograph of the cleaved end facet of a waveguide. Silicon is shown in green and sapphire in blue [79].

SOS wafers are primarily fabricated through epitaxial growth processes [79,80]. Since the device layer in SOS is silicon, their production shares similarities with SOI devices. Patterns on SOS wafers are typically created using dry etching with fluorine-based chemistries (e.g., CF_4_ [79], C_4_F_8_/SF_6_ [81]) or occasionally chlorine-based chemistries (e.g., Cl_2_/HBr/He [82]), akin to SOI wafer processing techniques. Initially, propagation losses in the SOS platform ranged from 4.3 to 4.9 dB/cm at 4.5 µm for a 1.8 × 0.6 µm ridge waveguide (Figure 3) [79]. Later advancements demonstrated that chemical oxidation followed by oxide stripping of silicon could significantly reduce losses to approximately 1 ± 0.3 dB/cm at 4 µm for a 2.4 × 0.48 µm nanowire waveguide.

#### 2.2.2. Germanium-Based Photonics

**Figure 4:**
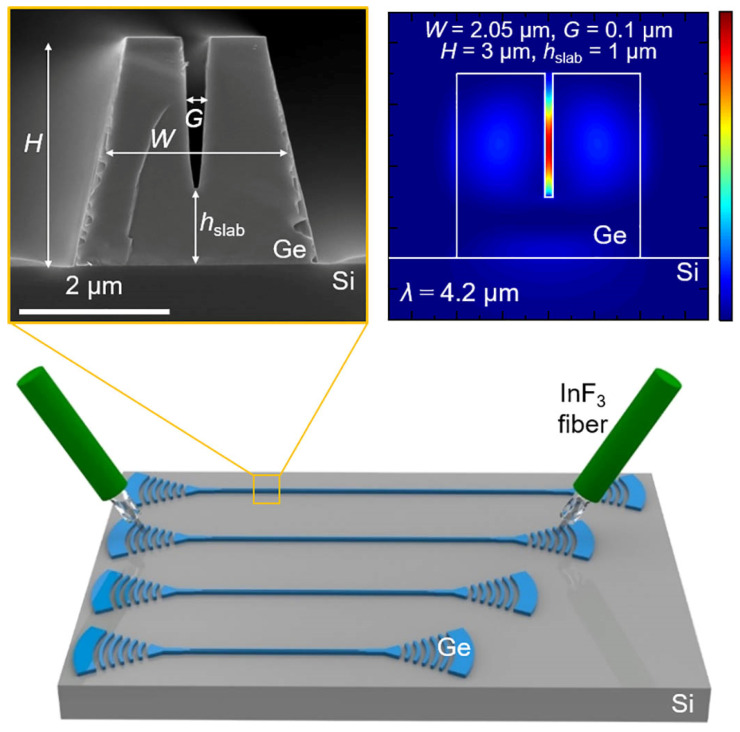
SEM image of the GOS waveguide with the 3 μm thickness of Ge slot waveguide. Experimental setup for measuring the propagation loss of waveguides [87].

Germanium-based platforms offer significant advantages due to their compatibility with CMOS technology and their broad transmission range of 1.8 µm to 14 µm. With a bandgap of 0.77 eV, germanium is well-suited for infrared photodetection and shows strong potential for monolithic integration [83], as it can be directly grown on Si(100) substrates [84,85]. Ridge waveguides based on germanium-on-silicon (GOS) for wavelengths around 5.8 µm were demonstrated using a 2 µm Ge layer, grown by reduced pressure chemical vapor deposition (RP-CVD) and patterned with a fluorine-based reactive ion etching (RIE) process. These waveguides exhibited propagation losses of 2.5 dB/cm and bending losses of 0.12 dB for a 90° bend with a 115 µm radius [86]. Another study described slot waveguides fabricated in a 3 µm thick Ge layer, etched with an inductively coupled plasma reactive ion etching (ICP-RIE) process (Cl_2_: 10 sccm, Ar: 30 sccm, RF power: 150 W, ICP power: 1000 W) using a nickel hard mask (Figure 4) [87]. The etching achieved core and slot depths of 3 µm and 2 µm, respectively, with a sidewall angle of 78°. The measured losses for the TE mode were 4.59–5.51 dB/cm in channel waveguides and 5.59–5.79 dB/cm in slot waveguides.

**Figure 5:**
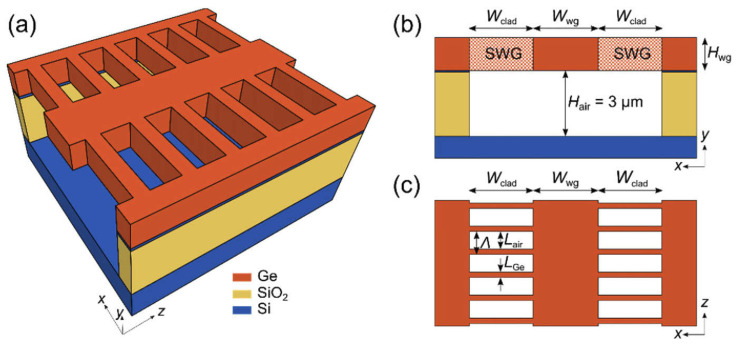
Schematics of a suspended germanium waveguide with subwavelength-grating metamaterial lateral cladding. (**a**) 3D view. (**b**) Front view (**c**) Top view of the guiding layer [88].

**Figure 6:**
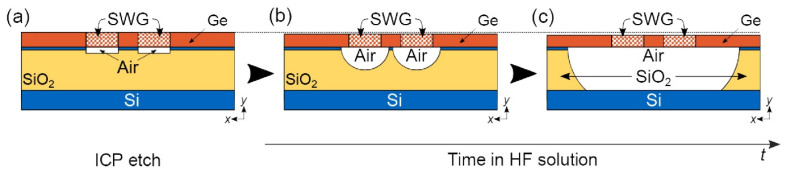
The process for fabricating suspended germanium waveguides with subwavelength grating lateral cladding involves multiple steps. (**a**) Dry etching is used to transfer the cladding holes from the photoresist to the germanium layer and silicon film. (**b**) The silicon dioxide and remaining silicon film are etched away using a wet etching technique. (**c**) The process concludes with the complete removal of the silicon film and buried oxide [88].

Suspending Ge waveguides effectively reduced propagation losses from over 60 dB/cm to 5.3 dB/cm at a wavelength of 7.7 µm [88]. The significant initial losses were likely attributed to the minimal separation between the SiO_2_ layer and the Ge waveguide, with only a 50–60 nm Si layer in between (Figure 5a–c). To prepare the structure, the SOI substrate (220 nm device layer, 3 µm BOX) was thermally oxidized and then wet-etched to reduce the Si layer thickness to 50–60 nm. A 1 µm thick Ge layer was subsequently deposited using RPCVD. The patterning process employed e-beam lithography, followed by ICP etching (SF_6_/C_4_F_8_ chemistry) down to the BOX layer. Waveguide suspension was accomplished by immersing the samples in a 1:7 HF:H_2_O solution for two hours (Figure 6a–c). Simulation results indicated that further loss minimization and extended propagation at wavelengths up to 15 µm could be achieved by optimizing the waveguide cross-section design.

#### 2.2.3. Two-Dimensional Materials

Two-dimensional (2D) materials have attracted considerable attention in recent years due to their unique properties and potential applications in various photonic and optoelectronic devices [89]. These materials, including black phosphorus (BP) [90] and transition metal dichalcogenides (TMDs) [91], offer exceptional flexibility, tunability, and light–matter interaction, making them particularly promising for mid-infrared (MIR) photonic sensors. The ability of 2D materials to exhibit both strong optical absorption and efficient electron transport in the MIR region presents significant advantages for applications like gas sensing, environmental monitoring, and biomedical diagnostics.

BP, one of the most notable 2D materials, has gained substantial interest for MIR sensing applications due to its highly tunable bandgap, which ranges from ~0.3 eV in bulk to ~2 eV in monolayers. This tunability allows for efficient light absorption in the MIR spectrum, particularly around the 4 to 5 µm range, where many molecular absorption features occur. BP also possesses strong in-plane anisotropy, which can be exploited in polarization-sensitive devices. Additionally, BP’s high carrier mobility (~1000 cm^2^/V·s) enables fast and efficient charge transport, making it an excellent candidate for applications that require rapid sensor response times. These unique characteristics make BP suitable for integration into photodetectors (PDs), modulators, and waveguides, enhancing the sensitivity and performance of MIR sensors [90,92].

Similarly, TMDs such as MoS_2_, MoSe_2_, WS_2_, and WSe_2_ exhibit remarkable optical and electronic properties that extend into the MIR range. TMDs feature strong excitonic absorption, even in the MIR regime, and can be engineered to achieve high absorption coefficients and tunable optical properties. These materials are particularly attractive for use in MIR PDs and modulators, where their high absorption and efficient light–matter interactions enable precise measurements and high sensitivity [93,94]. Moreover, TMDs can be integrated into silicon photonic platforms, making them an ideal choice for scalable, cost-effective MIR sensors. Their mechanical flexibility further adds to their versatility, allowing for the development of flexible and stretchable sensors for a wide range of applications, from wearable health monitors to environmental sensors [95].

#### 2.2.4. Other Materials

Indium phosphide (InP) is a semiconductor material that has gained significant attention in MIR photonics due to its favorable electronic and optical properties [96,97]. With a direct bandgap of 1.34 eV, InP offers efficient light absorption and emission in the MIR range, making it ideal for applications in MIR optoelectronics such as lasers, detectors, and modulators. Additionally, InP’s high electron mobility allows for fast device operation, which is beneficial for high-speed communication and sensing technologies [98]. The material’s compatibility with existing III-V compound semiconductors also enables the development of integrated photonic circuits, paving the way for compact and high-performance devices in applications like spectroscopy, environmental monitoring, and biomedical diagnostics. InP’s ability to operate efficiently in the MIR spectrum, along with its integration potential, positions it as a key material for advancing MIR photonic technologies [99].

Porous materials have emerged as promising candidates for MIR photonics due to their unique ability to engineer optical properties through controlled porosity and refractive index modulation [100]. These materials, including porous silicon, metal-organic frameworks (MOFs), and aerogels, offer tunable optical dispersion, low thermal conductivity, and a high surface area, making them ideal for applications such as infrared sensing, thermal imaging, and waveguiding [12,101,102]. Their porosity enables strong light–matter interactions, facilitating enhanced absorption, nonlinear optical effects, and tailored transmission characteristics in the MIR spectrum [103]. Furthermore, the ability to fabricate porous structures through techniques like electrochemical etching and templated synthesis provides additional flexibility in designing photonic devices with optimized performance in the MIR range [104,105].

**Figure 7:**
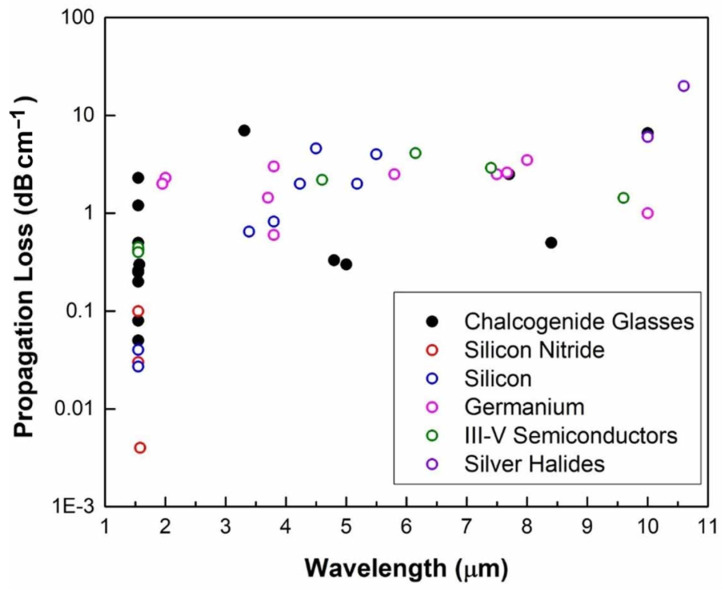
Waveguide propagation losses in various material platforms [107].

The advancement of ChG photonic devices is expected to persist [34,106], with a strong emphasis on minimizing optical losses for waveguide-based applications (Figure 7) [107]. This goal must be viewed in the broader context of alternative material platforms like SiN, which have achieved exceptionally low losses, reaching sub 1 dB/m. While ChGs have the inherent potential to match this performance—demonstrated by ultrahigh Q-factors of up to 70 million in ChG microspheres [108]—realizing similar low attenuation on integrated platforms requires substantial improvements in fabrication techniques. Another critical research area involves extending the operational wavelength range of ChG on-chip devices into the long-wave infrared (LWIR, 8–14 µm) and beyond. Although ChGs possess an unparalleled level of infrared transparency, distinguishing them from materials such as Si and SiN, most research has concentrated on near- and MIR applications, with only a few studies venturing into longer wavelengths [109].

Anne et al. explored the fabrication of chalcogenide fibers and planar waveguides. Using MIR transparent optical fibers, a biosensor for rapid, in situ metabolic analysis via remote infrared spectroscopy was developed [110]. The study examined spectral changes linked to transient focal ischemia in rat brains and starvation in mouse livers, using microdialysis as a reference. Additionally, reactive ion etching was employed to create rib waveguides (2–300 μm wide) and Y optical junctions on chalcogenide films, enhancing sensor sensitivity and stability. Initial functionalization tests were also conducted for potential (bio)sensor applications [110].

Moreover, as low glass transition temperature materials, ChGs belong to a class of ‘soft glasses’ that exist in a thermodynamically metastable state. Over time, or when subjected to moderate heat exposure—such as in backend processing or solder reflow during packaging—their structure can gradually relax, leading to shifts in optical properties. Addressing and mitigating these stability concerns through systematic characterization is crucial to ensuring reliable performance in photonic applications [107].

### 2.3. Integration Methods

In PICs, integration methods are essential for combining various photonic components, such as waveguides, modulators, and light sources, on a single chip or platform. Monolithic integration uses a single material, such as silicon or III-V semiconductors, to fabricate all components together, enabling high-density integration and efficient production, though material incompatibility can be a challenge [111]. Heterogeneous integration, on the other hand, combines different materials for specific functions, like using III-V materials for active components and silicon for passive ones, offering greater flexibility but introducing complexities in bonding and alignment [112]. Hybrid integration is a variation that combines photonic components sourced from different platforms, allowing for specialized functionality but often at a higher cost. Module-based integration assembles individual components into a larger package, offering flexibility but at the cost of a larger form factor [113]. 3D integration stacks multiple layers of devices, enabling a reduced footprint and high interconnection density but presenting challenges in heat dissipation and alignment [114]. Lastly, free-space optics and fiber integration allow for complex optical components and long-range communication but require precise alignment and add to system complexity. The choice of integration method depends on factors like performance requirements, material compatibility, cost, and scalability, with a growing trend toward heterogeneous and hybrid approaches for overcoming material limitations and achieving advanced system capabilities.

**Figure 8:**
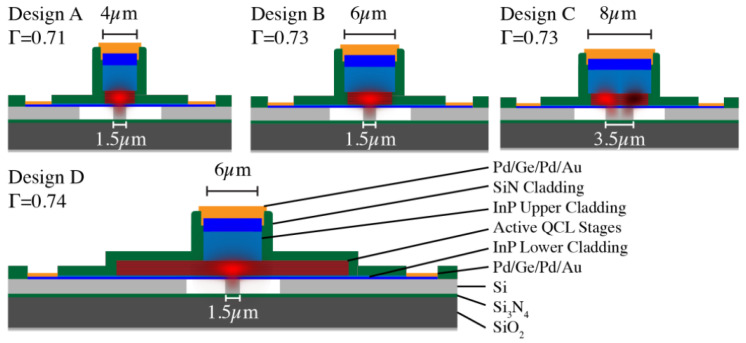
Cross-sectional schematics of the active regions for four different DFB QCL designs integrated on the SONOI waveguide [116].

An infrared thermopile sensor for broadband detection was integrated with MIR waveguides via flip-chip bonding and grating couplers [115]. This device, constructed on an SOI platform, was designed for N_2_O gas detection at 3.9 µm and achieved a resolution of 1800 ppm. A distributed feedback quantum cascade laser (DBF QCL) was also integrated heterogeneously onto silicon-on-nitride-on-insulator (SONOI) waveguides, which featured surface gratings etched through ICP (C_4_F_8_/SF_6_/Ar) [116]. Several configurations were explored (Figure 8), with lasers delivering over 200 mW of pulsed power and operating at wavelengths from 4.62 to 4.86 µm. In [117], a thermo-optic switch utilizing a graphene heater was developed and fabricated. The incorporation of graphene, a 2D material with minimal losses in the MIR spectrum, enabled a significant reduction in cladding thickness to just tens of nanometers, supporting direct heater placement on the waveguide. Demonstrated devices included an MRR switch with an 8 dB extinction ratio and rise/fall times of 3.72/3.96 µs, as well as a 2 × 2 MZI switch achieving an extinction ratio above 27 dB with rise/fall times of 4.92/4.97 µs.

### 2.4. Lithography Methods

The choice of lithography techniques is primarily dictated by the dimensions of the structures rather than the specific platform. Depending on the critical feature size, various approaches may be employed. For MIR waveguides, which typically have dimensions on the scale of a few micrometers, standard UV photolithography is sufficient [118] if submicron features are not required. For higher precision or smaller features, advanced techniques such as electron beam lithography (EBL), stepper-based UV lithography, or nanoimprint lithography (NIL) are necessary.

In research applications, EBL is widely utilized due to its ability to produce high-resolution patterns with smooth sidewalls [75,119]. Both negative-tone resists (e.g., HSQ [79], maN-2403 [120]) and positive-tone resists (e.g., ZEP-520A, AR-P 6200) are compatible with EBL. However, EBL is inherently expensive and time-intensive, which can limit its practicality for large-scale fabrication. The high cost of EBL arises from the need for specialized equipment, including electron beam writers, which are costly to purchase and maintain. Additionally, the process is slower compared to other techniques, making it less suitable for mass production and contributing to higher overall fabrication costs.

Stepper mask aligners, although less commonly used, can achieve submicron resolution and have demonstrated success in producing waveguides as narrow as 400 nm in silicon-on-sapphire platforms. They offer a faster alternative to EBL, but challenges arise when fabricating features such as narrow gaps in ring resonators. The high precision required for MIR sensors, which often involve very fine details, can make even advanced techniques like stepper lithography more complex and costly to implement, particularly when dealing with intricate designs that require multiple iterations or additional patterning steps.

NIL provides another viable approach for high-resolution fabrication. It enables the production of structures with low surface roughness and is particularly attractive for scalable manufacturing. When paired with appropriate stamps and material choices, NIL can function as a direct lithography method [121]. However, its adoption is constrained by the high cost of stamp fabrication and associated process complexities. The stamp-making process itself is highly specialized and expensive, and the integration of NIL into the production of MIR sensors requires advanced infrastructure, further escalating overall costs. NIL has been successfully applied in photonic device fabrication, including metamaterials [122], antireflective coatings [123], and optical filters [124].

Hybrid approaches can also be employed to optimize performance, combining the strengths of multiple techniques. For instance, EBL may be used to define high-precision features, while laser lithography or other lower-resolution methods can pattern less critical areas. This strategy balances the need for high resolution with the advantages of reduced time and cost [125]. However, even when combining techniques, the overall fabrication process for MIR sensors remains highly complex and costly due to the need for specialized materials, sophisticated equipment, and cleanroom environments to ensure the precision and reliability required for MIR performance. The intricate nature of these processes drives up the price of MIR sensors, limiting their widespread use and making large-scale manufacturing a significant challenge.

**Table 1:** Characteristics of material platform for MIR photonics.

Material Platform	Transparency Range (µm)	Refractive Index	Thermal Stability	Integration Compatibility	Key Advantages	Challenges
Silicon (Si)	~1.1–9.0	~3.4	High (up to 1200 °C)	CMOS-compatible	Mature fabrication technology, low loss in MIR	Absorption above 8 µm, difficult for high-power applications [20]
Silicon Nitride (Si_3_N_4_)	0.4–7.0	~2.0	High (~1200 °C)	CMOS-compatible	Low loss, easy to integrate with Si platforms	Limited transparency beyond 7 µm, lower index contrast [25]
Germanium (Ge)	2.0–14.0 [126]	~4.0	Moderate (~600 °C)	Compatible with Si platforms	Broad MIR range, high index contrast	Expensive, challenging for high-temperature applications [11]
Gallium Arsenide (GaAs)	0.9–17.0	~3.3	High (~700 °C)	Compatible with III-V platforms [127]	Wide transparency range, strong nonlinear properties	Expensive, complex integration [128]
Silicon Carbide (SiC)	0.5–15.0 [129]	~2.6	Very High (~1500 °C)	Compatible with Si platforms	High power handling, wide range	Limited CMOS compatibility, challenging fabrication [129,130]
Chalcogenide Glasses (ChG)	1.0–25 [131]	~2.2–2.8	Moderate (~400 °C)	Non-standard processes	Broad transparency, strong nonlinear properties	Low thermal stability, limited CMOS compatibility [131,132]
Indium Phosphide (InP)	1.0–12.0	~3.2	High (~600 °C)	Compatible with III-V platforms	High efficiency for light generation/detection [133]	High cost, challenging integration with Si [96,134]
Aluminum Nitride (AlN)	0.2–13.6 [135]	~2.0	Very High (~1800 °C)	CMOS-compatible	High thermal stability, low loss	Limited transparency in deeper MIR regions [136,137]
Calcium Fluoride (CaF_2_)	0.2–9.0 [138]	~1.4	Moderate (~600 °C)	Limited	Very low loss, wide transparency	Brittle, challenging to integrate with standard platforms [139]

Table 1 outlines various MIR materials, but a deeper analysis of their optical properties and thermal stability is essential for understanding their trade-offs in MIR sensor applications. Si and Ge are both widely used, but Ge is preferred for longer MIR wavelengths due to its broader transparency range (2–14 µm) compared to silicon (1.1–7 µm), which exhibits significant absorption beyond 7 µm [11,23]. Additionally, Ge’s higher refractive index (~4.0) enables stronger optical confinement, making it advantageous for compact MIR photonic devices [86]. However, Si has superior thermal stability (up to ~1200 °C), whereas Ge degrades at ~600 °C in air, limiting its use in high-temperature environments. Similarly, Si_3_N_4_ and ChGs present another trade-off; Si_3_N_4_ is highly thermally stable (~1200 °C) and CMOS-compatible but is only transparent up to 7 µm, while ChGs support a much broader MIR range (1–25 µm) but suffer from lower thermal stability (~400 °C). For III-V materials, GaAs and InP both offer high refractive indices (~3.2–3.3) and wide transparency ranges, with GaAs extending up to 13 µm, making it valuable for nonlinear optical applications. However, these materials present integration challenges, with InP being more commonly used for active optoelectronic devices such as MIR lasers. Ultimately, material selection in MIR sensors depends on balancing optical performance with thermal and fabrication constraints. Ge is preferred over Si for broader transparency and stronger optical confinement, while Si remains ideal for high-temperature applications. Similarly, ChGs excel in long-wavelength MIR sensing despite their low thermal stability, whereas Si_3_N_4_ is better suited for integrated photonics. These trade-offs highlight the need for careful material selection based on specific MIR sensing requirements.

## 3. Sensing Mechanisms and Techniques

To effectively utilize MIR sensing techniques, it is essential to understand the underlying mechanisms that drive their operation. Some of the most prominent sensing methods include absorption spectroscopy, evanescent wave sensing, and photoacoustic and photothermal techniques. Absorption spectroscopy, which encompasses methods such as Fourier-transform infrared (FTIR) [41,140] and tunable diode laser absorption spectroscopy (TDLAS) [141,142], allows for highly specific molecular absorption measurements. Evanescent wave sensing leverages surface-sensitive evanescent fields to detect molecular interactions occurring near interfaces [143,144,145]. Meanwhile, photoacoustic and photothermal techniques transform absorbed MIR radiation into measurable acoustic or thermal signals, facilitating the detection of trace amounts [146]. Additionally, emerging techniques like MIR fluorescence and nonlinear optical effects (e.g., difference frequency generation) offer enhanced sensitivity and precision, broadening the scope of MIR sensing in fields such as environmental monitoring, industrial applications, and biomedical research [147].

Detectors used in various optical and imaging techniques are essential for capturing and converting light or acoustic signals into measurable data. PDs are commonly used to detect light across different wavelengths, converting light into an electrical signal. These include devices like photodiodes, photomultiplier tubes (PMTs), and charge-coupled devices (CCDs), which are typically employed in applications such as spectroscopy, imaging, and light detection in scientific research [148]. PDs can be sensitive to a wide range of light, from UV to IR, depending on the material used. On the other hand, in photoacoustic techniques, piezoelectric transducers are primarily used to detect the acoustic waves generated by the rapid thermal expansion of a material after it absorbs pulsed light. These transducers convert sound waves into electrical signals for imaging or analysis, playing a key role in applications like medical imaging and material science [149]. Additionally, fiber-optic sensors and Capacitive Micromachined Ultrasonic Transducers (CMUTs) are emerging as important alternatives in these areas, offering enhanced sensitivity and resolution [150]. Each type of detector is selected based on the specific needs of the application, such as sensitivity, response time, and the nature of the signals being measured, allowing for tailored solutions in fields like biomedicine, material testing, and environmental monitoring.

### 3.1. Absorption Spectroscopy: Fourier-Transform Infrared (FTIR) and Tunable Diode Laser Absorption Spectroscopy (TDLAS)

Absorption spectroscopy, particularly Fourier-transform infrared (FTIR), is one of the most widely used techniques for analyzing samples with MIR light. First demonstrated in 1911 for biological sample analysis and further refined in the 1950s, FTIR is based on the ability of molecules to absorb infrared radiation at specific wavelengths associated with their unique vibrational transitions [151]. By examining the absorption spectra, FTIR offers both qualitative and quantitative insights into a sample’s chemical composition [152]. Advanced variations, such as attenuated total reflectance (ATR), extend FTIR’s capabilities, making it invaluable in fields like pharmaceuticals, biomedical research, and food quality control [153].

The key to FTIR’s sensitivity lies in its ability to detect the “fingerprint” of a molecule, as each substance has a distinctive absorption spectrum based on its molecular structure. The basic operation of FTIR involves passing infrared radiation through a sample, with an interferometer splitting and recombining the IR beam to create an interference pattern. The IR beam interacts with the sample, where absorption occurs at specific wavelengths corresponding to the functional groups of molecules. The unabsorbed radiation is then detected, typically using sensors like Mercury Cadmium Telluride (MCT) or Deuterated Triglycine Sulfate (DTGS). A Fourier-transform algorithm converts the data into a spectrum, which displays absorption peaks related to the molecular vibrations. This non-destructive, rapid technique provides detailed chemical information, enabling accurate identification and analysis of substances [154].

Jahromi et al. developed an innovative gas sensor that combines a compact, high-speed Fourier-transform spectrometer (FTS) with a broadband MIR supercontinuum (SC) source [155]. Spanning the entire spectral range of the SC source (2–4 µm), the system achieved an impressive spectral resolution of 1 GHz in just 6 s. It demonstrated exceptional detection sensitivity, reaching several hundred ppbv·Hz^−¹/₂^ for various gases. Key performance metrics, including precision, linearity, long-term stability, and multi-species detection capabilities, were thoroughly evaluated. The sensor was employed to analyze volatile compounds emitted by fruits under different atmospheric conditions and was compared with the performance of a previously developed scanning grating-based spectrometer [155].

Another prominent method for absorption spectroscopy is TDLAS, which is particularly effective in gas and plasma sensing. TDLAS works by directing a tunable diode laser through a gas sample and measuring the absorption of light at specific wavelengths that correspond to the molecular transitions of the target gas. The wavelength of the laser is scanned across an absorption feature, and the resulting decrease in light intensity, governed by the Beer–Lambert law, reveals the concentration of the gas. This technique provides highly sensitive, real-time measurements of gas properties such as concentration, temperature, and pressure. Advanced features like wavelength modulation further improve sensitivity, making TDLAS invaluable for applications in environmental monitoring, industrial processes, and gas analysis.

Food safety has become an increasingly critical focus in recent years, driven by the need to ensure the quality and safety of food products. Rapid detection methods for identifying contaminants in both the manufacturing process and final products have advanced significantly. Gruska et al. explored the use of FT-MIR to identify various contaminants in white sugar production [156]. The research highlighted the technique’s capability to accurately detect impurities, including inorganic compounds such as calcium carbonate (CaCO_3_), plastic materials like polypropylene, and oily substances from compressor sealing and lubrication systems. FT-MIR spectroscopy proved to be a reliable and efficient method for the rapid detection of sugar contaminants, even without reliance on complex spectral analysis techniques. Additionally, the incorporation of commercial reference spectra databases simplified and streamlined the application of this method, reinforcing its value for enhancing safety measures in sugar production.

**Figure 9:**
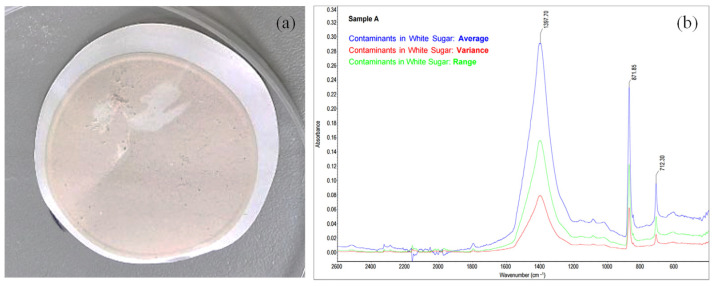
(**a**) Precipitate of the contaminant (Sample A) retained after filtering the white sugar solution [156], (**b**) statistical analysis of the FT-MIR spectra for contaminants in white sugar (samples analyzed: 3; total scans: 96). For each data point, the average represents the arithmetic mean of Y values, variance denotes the standard deviation of Y values, and range indicates the margin of Y values [156].

An external, water-insoluble contaminant (Sample A) was identified in white sugar. After dissolving the sugar in water and filtering the solution, a creamy-white precipitate remained on the filter surface (Figure 9a). Due to the complexity of determining the contamination’s nature and origin based solely on visual examination, MIR spectroscopy was employed. The precipitate was combined with KBr to form a pellet. The resulting spectra were analyzed statistically and the findings are depicted in Figure 9b [156].

### 3.2. Evanescent Wave Sensing (EWS)

EWS relies on the principle of total internal reflection, where light traveling through a high-refractive-index medium, such as an optical fiber or a waveguide, generates an evanescent field at the interface with a lower-refractive-index medium, penetrating a short distance into the external medium and decaying exponentially. This field interacts with molecules, enabling the detection of their unique absorption spectra, particularly in the MIR range (3–20 μm), where strong molecular vibrational bands provide detailed chemical information [157,158]. Techniques such as Fiber-Optic Evanescent Wave Spectroscopy (FEWS) combine optical fibers, often made of chalcogenide or silver halide, with Fourier-transform infrared (FTIR) spectroscopy to record these spectra [159,160]. Applications include environmental monitoring, where MIR-FEWS sensors detect pollutants like chlorinated hydrocarbons in seawater at ppb levels [157], and medical diagnostics, where FEWS has been used to identify biochemical markers in skin lesions for the rapid, non-invasive detection of melanoma, basal cell carcinoma (BCC), and squamous cell carcinoma (SCC) [160]. Additionally, chalcogenide fibers have been employed to analyze chemical solutions, such as acetone in water, demonstrating the versatility of EWS for real-time, in situ analysis across various domains [159].

The integration of photonic chips with electronic and micromechanical systems is poised to revolutionize laser spectroscopic sensors, enabling compact, precise, and reliable solutions for climate research and industrial applications. However, the sensitivity of chip-scale devices has been limited by challenges such as high waveguide losses, weak light–analyte interactions, and interference effects caused by chip facets and defects.

**Figure 10:**
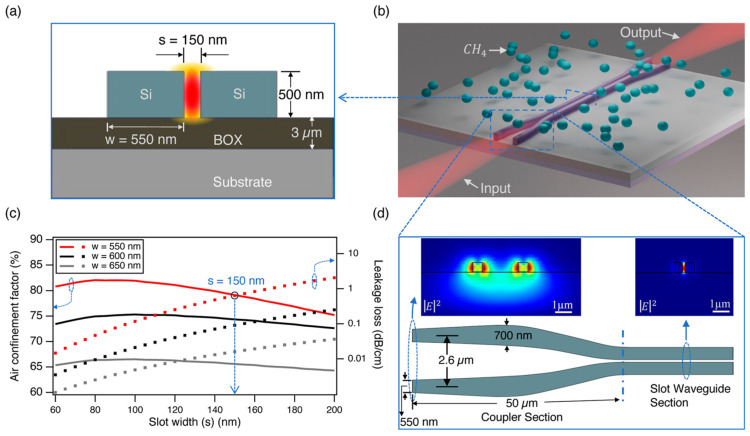
Key components of the sensor design: (**a**) the cross-sectional view of the slot waveguide [161], (**b**) the sensor chip layout featuring double-tip couplers, (**c**) simulation results showing the air confinement factor (solid lines) and substrate leakage loss (dotted lines) as functions of slot width for various strip widths, and (**d**) a schematic of the double-tip coupler, including simulated mode profiles at the coupler facet and slot waveguide cross-section [161].

To address these issues, Yallew et al. developed a nanophotonic waveguide specifically designed for methane detection at 3270.4 nm, achieving an extraordinary detection limit of 0.3 ppm—over two orders of magnitude better than current on-chip spectroscopy technologies [161]. Figure 10 highlights the design: (a) the cross-section of the slot waveguide and (b) the top-view layout of the sensor, modeled using Lumerical software to function on commercial SOI wafers. Ambient air serves as the top cladding and the gas interaction region. The waveguide design was optimized to support the fundamental TE mode, reduce leakage through the buried oxide layer (BOX), and maximize Γair. As illustrated in Figure 10c, Γair peaks broadly around a slot width of 90 nm. Considering fabrication constraints, the slot width was set at 150 nm and the waveguide core width at 550 nm, yielding Γair = 79% with leakage losses under 1 dB/cm.

To further enhance performance, double-tip couplers (Figure 10d) were integrated at both ends of the waveguide, improving coupling efficiency and suppressing reflections. This innovation was made possible through a silicon slot waveguide optimized for robust light–analyte interactions, combined with advanced double-tip fork couplers that effectively eliminated unwanted etalon fringes at the waveguide facets [161].

### 3.3. Photoacoustic and Photothermal Techniques

Photoacoustic sensing is based on the generation of acoustic waves through the absorption of modulated light energy by a sample, where the absorbed light energy is converted into heat via non-radiative relaxation, producing pressure waves detectable by a microphone. The principle relies on modulating the light source—commonly lasers or LEDs—to create periodic heating and cooling in the sample, generating sound waves proportional to the sample’s absorption characteristics. The technique can be enhanced by using non-resonant photoacoustic cells for compact designs and resonant cells for enhanced sensitivity, as used in an MIR fiber-coupled sensor for detecting glucose in biomedical applications [162]. Another example is a miniature MIR photoacoustic gas sensor, which uses a MEMS thermal light source and a PDMS membrane for detecting dissolved CO_2_ in seawater with a detection limit of 0.72 ppm and a fast response time of 3.5 min [163]. Additionally, a low-cost photoacoustic CO_2_ sensor can be designed using an MIR LED to excite sound waves in a hermetically sealed chamber, demonstrating a scalable approach for environmental monitoring [164]. These examples highlight the adaptability of photoacoustic sensing in biomedical diagnostics, environmental monitoring, and gas detection.

Photothermal sensing is a highly sensitive technique that measures the absorption of light by a sample and its conversion to heat, leading to localized changes in temperature, refractive index, or pressure [165]. The principle involves the use of a pump laser to excite the sample and induce these changes, which are then detected by a probe light via shifts in optical properties or interference patterns. Detection can be enhanced using approaches such as hollow-core fibers, which confine light and gas interactions to achieve high sensitivity, or microring resonators, which use the thermo-optic effect to transduce MIR absorption into measurable optical changes [166]. Techniques like MIR Photothermal (MIP) Microscopy offer label-free imaging of biological and material samples by exploiting vibrational absorption-induced thermal effects, enabling high spatial resolution and minimal interference from water absorption [167]. Applications span from trace gas detection, such as nitrous oxide monitoring at sub-ppm levels [165], to chemical imaging of polymers and cellular structures [166,167], making photothermal sensing versatile in environmental monitoring, biosensing, and material science.

**Figure 11:**
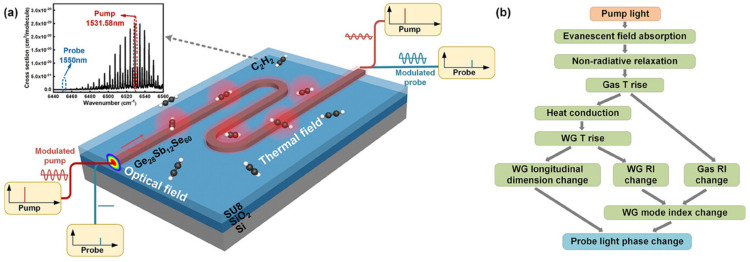
(**a**) Diagram of the on-chip PTS setup with a modulated pump, where the phase of the probe beam is altered due to pump absorption. The inset displays the C_2_H_2_ absorption cross-section at the pump and probe wavelengths [168]. (**b**) Illustration of the processes responsible for PT-induced phase modulation in the waveguide [168].

Zheng et al. introduced on-chip photothermal spectroscopy (PTS) as a powerful technique for gas detection, achieving exceptional sensitivity and an extended dynamic range [168]. The study meticulously analyzed the photothermal field, generated through the non-radiative relaxation of gas molecules, and its resulting phase modulation. To optimize thermal field accumulation, ChG was selected as the core material, and thermally isolated ChG-on-SU8 waveguides were designed. This design enhanced photothermal phase modulation by a factor of two compared to ChG-on-SiO_2_ waveguides (Figure 11a). Unlike DAS, which is often limited by multi-path etalon noise, PTS was primarily influenced by piezoelectric transducer noise within the interferometer. For comparison, acetylene (C_2_H_2_) detection experiments were conducted using both PTS and DAS with a 2 cm-long ChG-on-SU8 waveguide. PTS delivered an impressive detection limit of 4 ppm, outperforming DAS by a factor of 16. Moreover, its dynamic range spanned five orders of magnitude, approximately three times broader than that of DAS [168].

Figure 11b illustrates the process and key factors involved in the on-chip PT-induced probe phase modulation. In a hollow-core fiber (HCF), the variation in the probe phase was mainly influenced by the thermal optical coefficient (TOC) of the gas medium inside the core. In a waveguide, significant enhancement of PT phase modulation can be achieved by utilizing a strong pump field near the waveguide interface, materials with high TOC and thermal expansion coefficients (TEC), and selecting an appropriate probe wavelength. The resulting phase modulation can be effectively detected using an optical interferometer, such as a Mach–Zehnder interferometer (MZI), with active homodyne stabilization. This outstanding performance opens the door to fully integrated chip-level sensors for low-power, lightweight applications [168].

### 3.4. Fluorescence and Nonlinear Techniques

The above-mentioned methods cover the most common techniques; however, there are also other techniques worth looking at. Some of them involve fluorescence and luminescence effects but others also use nonlinear effects.

Fluorescence sensing involves detecting specific substances by measuring the emission of light (fluorescence) from a material, often after excitation by a light source [169,170,171]. In MIR fluorescence sensing, materials such as Dy^3+^-doped Ga_5_Ge_20_Sb_10_S_65_ glass fibers emit light at specific wavelengths when excited, leveraging their unique fluorescence properties to overlap with absorption bands of target molecules like CO_2_. This technique relies on a differential measurement principle, comparing the intensity of emitted light within and outside the absorption band of the target molecule to determine its concentration with high sensitivity and resolution. For example, Dy^3+^-doped fibers, excited with a laser at 920 nm, emit fluorescence around 4.35 µm, which overlaps with the CO_2_ absorption band, enabling atmospheric and geological CO_2_ monitoring [172]. Similarly, in single-molecule spectroscopy, plasmonic nanocavities enhance fluorescence by coupling vibrational and electronic transitions, allowing MIR photons to be upconverted into visible wavelengths for highly sensitive molecular detection [41]. These approaches demonstrate the versatility of fluorescence sensing for applications in environmental monitoring, gas detection, and molecular-level spectroscopy. Nevertheless, it is worth mentioning that also taking leverage of nonlinear effects can be beneficial for sensing applications [173].

## 4. Types of MIR Photonic Sensors

This section explores two distinct types of MIR photonic sensors: waveguide-based sensors and fiber-based sensors. Each offers unique advantages and applications in the field of photonic sensing.

### 4.1. Optical Waveguide-Based Sensors

Waveguides play a crucial role in the design of PICs, irrespective of the material platform or the spectral range they operate within [174,175,176]. Beyond their primary function of guiding light, they also serve as sensors or as media for nonlinear interactions. Effective light guiding in waveguides typically demands minimal optical power losses and compact bending radii, which facilitate higher packing densities in photonic circuits [73,177]. When used as sensors, waveguides must enable the guided optical signal to interact with the target medium—be it gas, liquid, or solid [28,178,179,180]. This interaction is achieved through the evanescent field, a portion of the optical signal that extends into the interface between the waveguide and its surroundings [143,181]. Using waveguides with thicknesses smaller than the propagating wavelength can establish a consistent evanescent field, leading to improved sensitivity compared to other sensor types, such as those based on optical fibers or bulk elements leveraging total internal reflection. Enhancing the interaction between the waveguide and the material being measured involves increasing the penetration depth of the evanescent field. 

Optical waveguide sensors operating in the MIR wavelength range are of significant importance due to their ability to exploit the strong molecular absorption features characteristic of many chemical and biological species in this spectral region [26,29]. The MIR range, often referred to as the “molecular fingerprint region”, allows for highly selective and sensitive detection of gases, liquids, and solids by directly probing their unique absorption lines [182]. These sensors are particularly valuable in environmental monitoring, enabling the precise detection of greenhouse gases such as CO_2_, CH_4_, and N_2_O [183,184,185]. They are also critical in industrial process control, where real-time monitoring of volatile organic compounds (VOCs) enhances safety and efficiency [186]. Furthermore, MIR waveguide sensors are advancing medical diagnostics through non-invasive analysis of biomarkers in human breath and body fluids. Their compactness, robustness, and integration potential make them ideal for portable sensing devices, opening new possibilities in remote sensing, homeland security, and food quality assurance.

**Figure 12:**
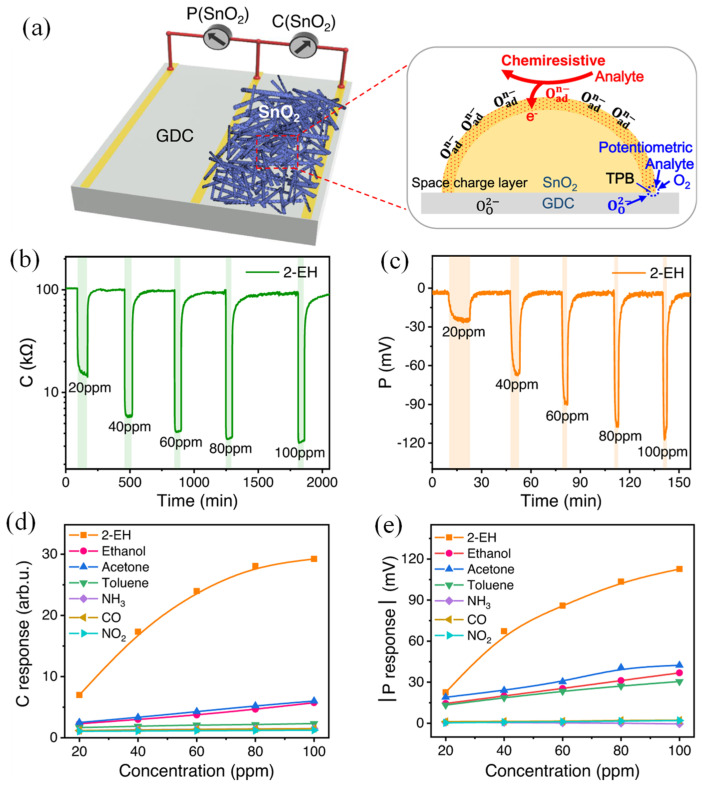
(**a**) Diagram of the proposed multivariable sensor, showing the chemiresistive-potentiometric sensing region. (**b**) Chemiresistive and (**c**) potentiometric (relative to Pt CE) response times for various 2-EH concentrations. (**d**) Chemiresistive and (**e**) potentiometric responses to 2-EH, ethanol, acetone, toluene, NH_3_, CO, and NO_2_ at different concentrations. The sensor operates at 400 °C [187].

Efficient gas sensors are essential for detecting hazardous gases, but conventional single-output sensors face issues like drift, size, and cost. Zhang et al. presented a sensor combining chemiresistive and potentiometric outputs, compatible with various electrodes and solid electrolytes for customizable sensing [187]. Enhanced with a mixed-conducting perovskite electrode, it demonstrated exceptional sub-ppm sensitivity, distinguishing humidity from seven hazardous gases (2-Ethylhexanol, ethanol, acetone, toluene, ammonia, carbon monoxide, and nitrogen dioxide), while providing early fire hazard warnings. C-P gas sensing using a SnO_2_-based SE on a GDC solid electrolyte substrate was presented, as shown in Figure 12a. The C signal was caused by changes in resistance due to the interaction of analyte gases with oxygen on the SE surface, while the P signal (SnO_2_-Pt) arose from potential changes at the gas/SE/electrolyte three-phase boundary. Figure 12b,c display the dynamic response of SnO_2_ to seven gases, including four VOCs (2-Ethylhexanol, ethanol, acetone, toluene) and three inorganic gases (NH_3_, CO, NO_2_). When exposed to reducing (oxidizing) gases, both resistance and potential decrease (increase), exhibiting typical n-type and non-Nernstian sensing behavior, SnO_2_ showed strong responses to 2-EH, a marker for indoor air pollutants and fire hazards, while responses to NH_3_, CO, and NO_2_ are weaker (Figure 12d,e). The responses to other VOCs were moderate for P, with C responses smaller than for 2-EH. These differences indicate that the sensing mechanisms are independent.

An MIR hollow waveguide gas sensor was developed to detect the absorption lines of H_2_^18^O, H_2_^16^O, H_2_^17^O, and HDO at 3662.9196, 3663.04522, 3663.32128, and 3663.84202 cm^−1^, respectively [188]. The sensor utilized wavelength modulation spectroscopy with a 2.73 µm distributed feedback diode laser. Gas absorption was measured using a 5 m long hollow waveguide fiber with a 1 mm inner diameter. Calibration was performed using a dew point generator containing liquid water with known water/isotope ratios. Detection limits achieved were 35.18 ppbv for H_2_^18^O, 4.69 ppmv for H_2_^16^O, 60.53 ppbv for H_2_^17^O, and 3.88 ppbv for HDO with a 96 s integration time. This allowed for isotopic ratio measurement precisions of 0.85‰ for δ^18^O, 0.57‰ for δ^17^O, and 10.48‰ for δD. Field tests for H_2_^18^O, H_2_^16^O, and H_2_^17^O concentrations were carried out at the Nanchang Hangkong University campus to assess the sensor’s performance [188].

Subwavelength grating (SWG) metamaterial sensors are designed using periodic structures with minute dimensions that still enable light propagation [14,189]. Employing a periodic arrangement of pillars, which permits the analyte to enter the gaps, enhances the interaction between the light mode and the surrounding material under examination. This design significantly increases the sensitivity of the sensor element [76]. Achieving optimal sensitivity in SWG metamaterial structures requires careful geometric design. In addition to enhanced sensitivity, SWG elements offer other advantages, such as the ability to modify waveguide dispersion, isolate the fundamental mode, and control the optical bandwidth [190,191]. Two critical parameters for achieving these functionalities are the grating period and the duty cycle, defined as the ratio of the individual pillar length to the period. To prevent Bragg reflection, the grating period must be smaller than the Bragg period. Furthermore, the period should be significantly reduced to ensure operation in the subwavelength regime [192]. 

An MIR SWG coupler and suspended membrane waveguide (SMW) on a silicon-on-insulator wafer were investigated. For a transverse-electric mode uniform SWG, finite-difference time-domain simulations predicted a coupling efficiency of 44.2%, a 1 dB bandwidth of approximately 220 nm, and a backreflection of 0.78% at a wavelength of 2.75 μm. The uniform SWG was then modified into a focusing SWG using a phase-matching formula. The SMWs were analyzed using the finite element method and subsequently fabricated. A co-doped MIR fiber laser was employed for device characterization, and the fabricated MIR SWG coupler achieved a coupling efficiency of 24.7% [193].

**Figure 13:**
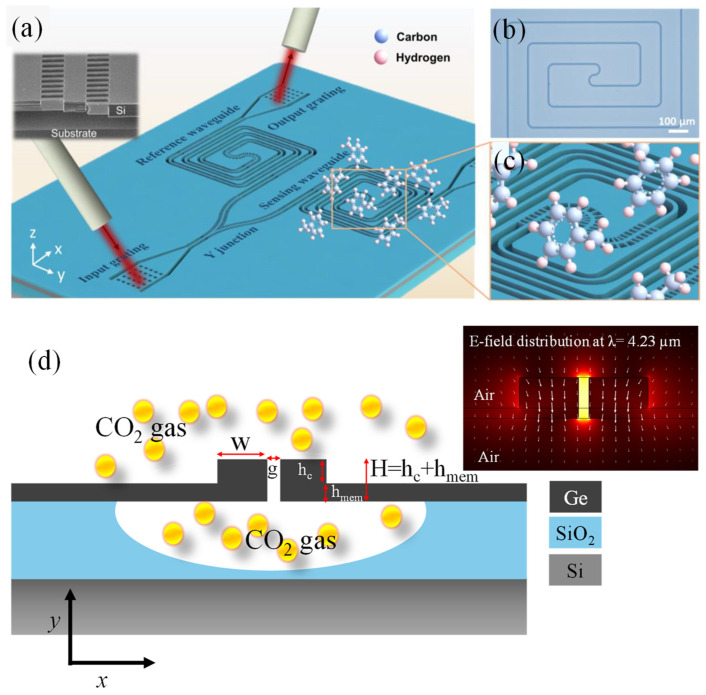
(**a**) Diagram of the suspended Si waveguide gas sensing platform, which includes grating couplers, tapers, a Y-junction power splitter, and spiral waveguides. Inset: Cross-sectional SEM image of the waveguide [194]. (**b**) Optical image of the suspended Si spiral waveguide [194]. (**c**) Close-up view of the sensing waveguide surrounded by toluene molecules, highlighted by the yellow box in (**a**) [194]. (**d**) Schematic of suspended slot membrane waveguide based on Ge-on-SOI platform for CO_2_ gas detection. The inset shows the E-field distribution at an operational wavelength of 4.23 µm [195].

Liu et al. presented a long wave-infrared (LWIR) photonic platform for rapid and sensitive on-chip gas sensing, utilizing suspended silicon waveguides supported by subwavelength grating (SWG) metamaterial claddings (Figure 13a) [194]. Figure 13b presents an optical microscope image of the spiral structure used in the experiment, featuring a sensing length of 28.4 mm. Figure 13c provides a close-up view of the spiral sensing area. In this setup, toluene molecules were evenly distributed around the waveguide, including the SWG cladding and the upper and lower air claddings, where they interacted with the evanescent field of the guided light, leading to additional absorption. This approach effectively leveraged the transparency window of silicon while the SWG structure optimized the mode profile for enhanced light–analyte interaction. Propagation and bending losses were analyzed across the 6.4–6.8 µm wavelength range. High-performance functional devices, including grating couplers, Y-junctions, and directional couplers, were demonstrated. The platform’s sensing capability was showcased through toluene vapor detection, achieving a detection limit of 75 ppm with response and recovery times of approximately 0.8 and 3.4 s, respectively. These results highlighted the platform’s potential for on-site medical and environmental applications [194]. 

Recently, Butt et al. proposed an innovative suspended slot membrane waveguide designed on a germanium-on-silicon-on-insulator (Ge-on-SOI) platform, specifically for detecting CO_2_ gas (Figure 13d) [195]. The proposed structure was tailored to operate at the CO_2_ absorption wavelength of 4.23 µm in the MIR spectrum. The waveguide’s geometry was meticulously engineered to maximize the evanescent field ratio (EFR) while minimizing propagation losses, enhancing its suitability for evanescent field absorption-based gas detection. The optimized design achieved a high EFR of 0.86, a propagation loss as low as 1.07 dB/cm, and exceptional sensitivity of approximately ~1.12 × 10^−4^ ppm^−1^ with a compact SSMW length of only 0.9 cm.

Plasmonic waveguide-based sensors for MIR applications represent a cutting-edge approach to the highly sensitive detection of molecular fingerprints and environmental monitoring [196,197]. These sensors leverage surface plasmon polaritons (SPPs)—electromagnetic waves coupled to electron oscillations at metal–dielectric interfaces—to confine and guide light at subwavelength scales. Plasmonic waveguides, designed with materials like gold, silver, or emerging MIR-compatible alternatives (e.g., graphene or doped semiconductors), enhance light–matter interactions, thus improving detection limits [42,198]. Their compact size, tunable response, and compatibility with integrated photonic circuits make them ideal for developing portable and efficient sensing platforms in applications ranging from environmental monitoring to medical diagnostics [199].

**Figure 14:**
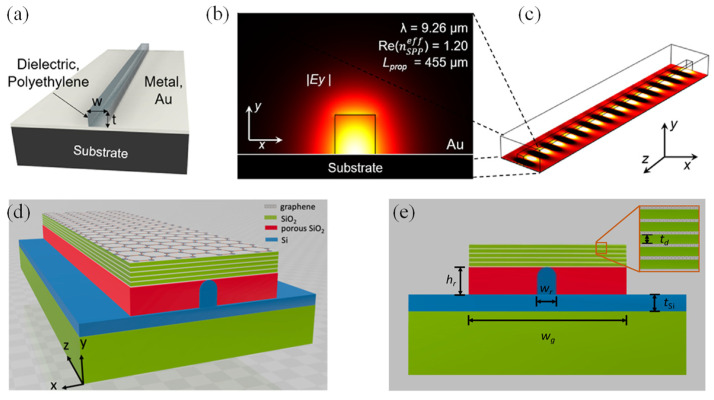
(**a**) Schematic of a DLSPP waveguide [196]. (**b**) Cross-sectional view and (**c**) top view of the E-field distribution in the waveguide [196]. (**d**) 3D representation and (**e**) cross-sectional view of MLGMT waveguide [200].

David et al. proposed LWIR/MIR PICs by integrating photolithographic patterning of organic polymers with dielectric-loaded surface plasmon polariton (DLSPP) waveguides (Figure 14a) [196]. This configuration comprised a dielectric ridge positioned above a metallic layer. The cross-sectional and top-view of the E- field distribution for a polyethylene ridge is depicted in Figure 14b and Figure 14c, respectively. Notably, polyethylene exhibits advantageous optical characteristics, such as a low refractive index and extensive transparency spanning from the MIR range to 200 µm. The entire development process was explored, covering the design, fabrication, and analysis of polyethylene-based DLSPP waveguides, highlighting their plasmonic behavior and mode-guiding performance in -bend configurations for the first time. These waveguides demonstrated minimal bending losses and enable propagation over significant straight-section lengths, setting the stage for intricate on-chip MIR photonic systems. Additionally, DLSPPs provided precise control over mode properties, including propagation length and guiding efficiency, making them suitable for advanced applications in sensing and telecommunications through compact, chip-scale devices.

Graphene-based plasmonics, which can confine MIR light beyond the optical diffraction limit, present a promising approach for photonic chip integration. However, existing designs are limited to propagation lengths of approximately 10 µm at a working frequency of 20 THz. Huang et al. introduced a waveguide structure utilizing multilayer graphene metamaterials (MLGMTs) that support the fundamental volume plasmon polariton mode by coupling plasmon polaritons across graphene sheets on a silicon nano-rib structure [200]. The 3D view and cross-sectional view of the waveguide is shown in Figure 14d and Figure 14e, respectively. Due to the high conductivity of MLGMTs, the guided mode exhibited significantly lower loss compared to conventional graphene-based plasmonic waveguides with similar mode sizes. The proposed design achieved propagation lengths of around 20 µm—four times the current limitation—while maintaining a mode area as small as 10^−6^ A_0_, where A_0_ represents the diffraction-limited mode area. Detailed investigations of the modal characteristics based on geometric and material parameters were conducted to optimize device performance. The robustness of the structure was also evaluated against fabrication imperfections. Furthermore, crosstalk analysis between adjacent waveguides confirmed strong mode confinement, enabling high-density on-chip integration. This design provided a viable approach for developing tunable, large-area photonic integrated circuits [200].

### 4.2. Optical Fiber-Based Sensors

MIR optical fiber-based sensors have garnered significant attention due to their capability to operate in the MIR spectral range, which is highly suited for applications requiring high sensitivity and specificity [201]. Optical fibers designed for this spectral range serve as efficient waveguides, enabling the remote delivery and collection of MIR radiation [202]. Unlike conventional free-space systems, fiber-based sensors offer enhanced flexibility, compactness, and compatibility with complex environments, paving the way for their use in harsh industrial settings, medical diagnostics, and environmental monitoring [203,204].

The performance of MIR fiber-based sensors depends significantly on the material properties of the fiber. Traditional silica fibers are unsuitable for this range due to their high attenuation beyond 2.4 µm. Instead, specialized materials such as ChGs, fluoride glasses, and hollow-core fibers have emerged as leading solutions [205,206]. Chalcogenide fibers, composed of elements like sulfur, selenium, and tellurium, exhibit excellent transparency and robustness in the MIR spectrum, making them ideal for chemical sensing [207]. Fluoride fibers, on the other hand, provide lower losses in the short-wave MIR region, catering to applications requiring minimal signal attenuation. Hollow-core fibers, which guide light through air or gas-filled cores, offer even broader transmission windows with reduced material absorption, making them suitable for high-power and long-distance sensing [208].

MIR fiber-based sensors are particularly valuable in detecting trace gases, hazardous chemicals, and biomolecules, owing to the strong molecular fingerprints in the MIR range. They are widely employed in spectroscopy-based systems, where the fiber enables precise light delivery and sample interrogation. Innovations in fiber fabrication techniques and integration with microfluidics and photonic chips have further expanded their application scope, enabling real-time monitoring in fields like healthcare diagnostics, where non-invasive blood glucose sensing and cancer biomarker detection are gaining traction. Similarly, environmental monitoring benefits from their ability to detect pollutants like methane and carbon dioxide with high accuracy [209]. Despite their promise, MIR optical fiber-based sensors face challenges such as fabrication complexity, fragility of advanced materials, and high costs. Research efforts are focused on improving the durability and scalability of these fibers while reducing manufacturing expenses. With continuous advancements in materials science and integration technologies, the future of MIR optical fiber-based sensors looks bright, promising transformative applications across multiple domains [210,211].

**Figure 15:**
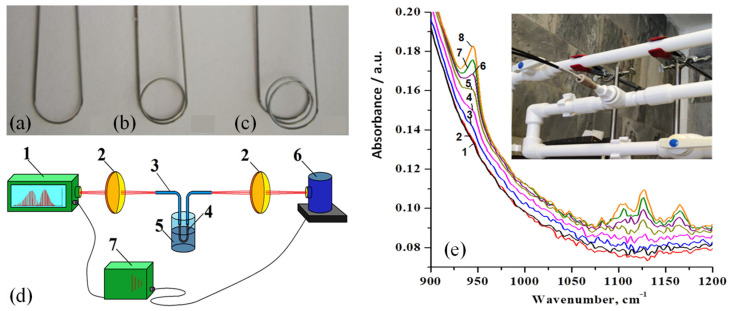
Various configurations of the probe’s sensing zone: (**a**) U-shaped; (**b**) single loop; (**c**) double loops. (**d**) Configuration for fiber-optic evanescent wave spectroscopy (FEWS) analysis: 1—IR Fourier spectrometer; 2—ZnSe focusing lenses; 3—chalcogenide fiber with polymer coating; 4—uncoated fiber sensing segment; 5—container with the liquid sample; 6—HgCdTe detector; 7—amplifier. (**e**) Image of the experimental setup used for in situ sensor testing. Absorption spectra of isopropyl alcohol in water–alcohol mixtures: 1—pure water; 2—0.1% vol. isopropyl alcohol; 3—0.5% vol.; 4—1.0% vol.; 5—2.0% vol.; 6—3.0% vol.; 7—4.0% vol.; 8—5.0% vol [212].

Recently, Shiryaev et al. highlighted the recent advancements in passive and active optical waveguides crafted from high-purity ChGss, tailored for MIR fiber optic evanescent wave spectroscopy of liquids and gases [212]. Innovative and highly sensitive fiber probes were designed using selenide and telluride glass fibers (Figure 15a–c) connected to the IR Fourier spectrometer using a lens system (Figure 15d). Additionally, radiation sources emitting in the 4.2–6 μm wavelength range were developed using Pr(3+)- and Tb(3+)-doped Ga(In)-Ge-As-Se and Ga-Ge-Sb-Se glass fibers, enabling their application in all-fiber sensor systems [212].

In industrial production settings, one of the key challenges is the real-time measurement of liquid chemical composition directly within the flow stream. To explore the feasibility of such measurements using fiber probes, a pipeline setup was developed. This system comprised soldered polypropylene pipes equipped with valves for flow rate control. A submersible pump was used to maintain a liquid mixture flow at a linear velocity of 0.5–1 m/s. The fiber-optic sensor was securely installed into the pipeline using a threaded plug and PTFE seal (Figure 15e, inset). The test liquid was a “water–isopropyl alcohol” mixture with alcohol concentrations ranging from 1 to 10 vol.%. The sensor demonstrated high sensitivity, linearity in its analytical signal, and robustness under turbulent flow conditions, highlighting its potential for in-line monitoring of liquid chemical compositions in industrial processes [212].

## 5. Applications of MIR Photonic Sensors

**Figure 16:**
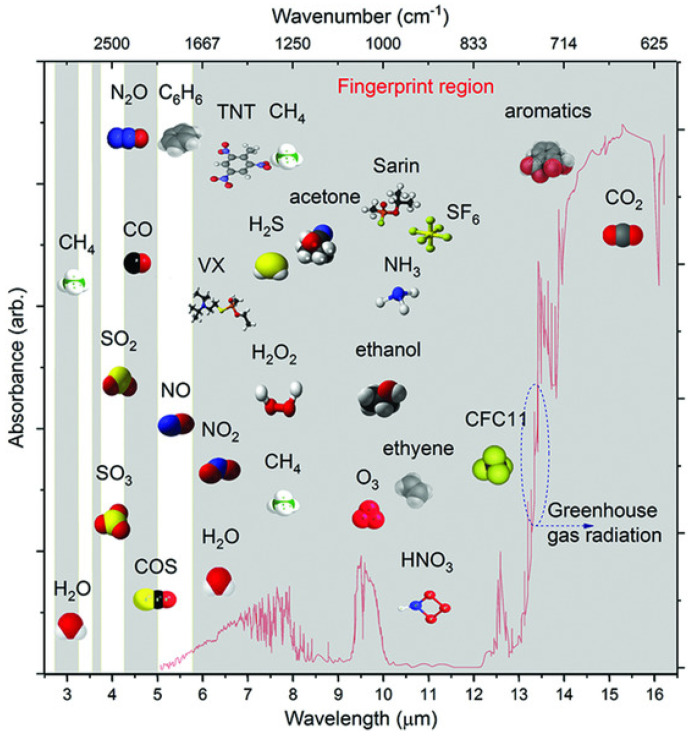
MIR spectrum with marked specific molecules absorption regions [213].

The MIR region offers significant potential for applications in biosensing, gas detection, medical diagnostics, environmental monitoring, and more. Figure 16 represents the selected material’s absorption peaks in the MIR range [213].

### 5.1. Environmental Monitoring

Environmental monitoring is essential for understanding and addressing the challenges of greenhouse gases, air pollutants, and water contaminants [184]. One of the tools that may be used for such applications is optical sensors operating on the MIR region. Regarding detecting greenhouse gases, MIR sensors exceed in the detection of CO_2_. The accelerating climate change is a big problem for the environment. CO_2_ should be monitored not only in the air but also in underground storage systems. Researchers developed methods for continuous environmental monitoring. One uses rare-earth-doped ChGss as optical materials for MIR environmental sensors. These glasses, doped with Pr^3+^ and Dy^3+^ ions, exhibit luminescence properties that match the CO_2_ absorption band at 4.3 µm. ChGs exhibit high IR transmission (up to 25 µm) and low non-radiative relaxation, improving sensor efficiency. It has been demonstrated that with these materials, a sensitivity of 15 ppmv can be achieved [214].

Moreover, Butt et al. presented a suspended slot membrane waveguide based on a Ge-on-SOI platform for CO_2_ gas sensing [195]. The design targeted the CO_2_ absorption line in the MIR spectrum at 4.23 µm, with a geometry optimized to maximize the evanescent field ratio (EFR) while minimizing propagation losses. These enhancements significantly improved sensitivity for evanescent field absorption-based gas detection. The optimized waveguide achieved an EFR of 0.86, a low propagation loss of 1.07 dB/cm, and a high sensitivity of approximately 1.12 × 10^−4^ ppm^−1^ for SSMW lengths as short as 0.9 cm.

In addition, regarding gas monitoring, it is worth mentioning the possibility of detecting ammonia and nitric oxide by using MIR wavelengths. Atmospheric monitoring of urban pollutants, including NH_3_ and NO, is critical for understanding air quality. Presented solutions use techniques like photoacoustic spectroscopy (PAS) and quartz-enhanced PAS (QEPAS), that allow even results of ppb resolution to be achieved. All these sensing devices operate on quantum cascade (QC) and interband cascade (IC) lasers [215]. 

Concerning water contamination, MIR Attenuated Total Reflectance (ATR) sensors deserve to be highlighted. The detection of organic pollutants, particularly hydrocarbons, in aqueous environments can be carried out by this method and achieve detection limits of 10–100 ppb in the concentration range. By using MIR ATR, it is possible to detect aromatic compounds, alkyl halides, and phenols in oceans, lakes, and rivers [46]. 

### 5.2. Industrial Quality Control and Safety

MIR sensors play a critical role in industrial and chemical process control, offering unmatched capabilities for detecting gases, liquids, and solids. Their high sensitivity, selectivity, and robustness make them indispensable tools for maintaining quality and safety standards across diverse industrial sectors. The integration of MIR sensors into industrial systems enhances process safety and minimizes environmental risks by enabling continuous the monitoring and early detection of hazardous leaks or contaminants. Gases such as CO, CO_2_, CH_4_, and SO_2_ exhibit strong fundamental absorption features in the MIR range, which are orders of magnitude stronger than their NIR overtones, and due to their distinct spectra of different molecules, the cross interference is minimized [216]. An exemplary gas sensor presented by Dong et al. can be built by using a single broadband light source covering the absorption bands of CO (4.65 µm), CO_2_ (4.26 µm), and CH_4_ (3.31 µm) and multiple pyroelectric detectors mounted on a rotating disc controlled via a stepper motor, enabling sequential gas detection. It could find applications in coal mine safety, where monitoring gases like CO, CO_2_, and CH_4_ is critical to prevent accidents and ensure worker safety. The presented sensor achieved detection limits of 2.96 ppmv (CO), 4.54 ppmv (CO_2_), and 2.84 ppmv (CH_4_) [217].

Beneitez et al. introduced a novel evanescent wave sensing platform operating in the 6.5 to 7.5 µm wavelength range, demonstrated for the detection of toluene in aqueous solutions [218]. The system featured a Ge-on-Si waveguide with a hydrophobically functionalized mesoporous silica cladding and integrated microlenses for alignment-tolerant optical coupling to a tunable laser spectrometer. The functionalized cladding enhanced the enrichment of apolar analytes while minimizing water interference with the evanescent wave. Performance evaluation with aqueous toluene standards achieved an LOD of 7 ppm. Adsorption and desorption profiles followed Freundlich isotherms, exhibiting rapid equilibration and response times of just a few seconds, highlighting its potential for real-time water quality monitoring. Further improvements in LOD are expected with reductions in spectrometer noise, currently at a relative standard deviation of approximately 10^−2^ A.U [218].

### 5.3. Biomedical and Clinical Diagnostics

It is also worth mentioning that the MIR wavelength region can also be useful in other sensing applications than environmental monitoring. Biomedical and clinical diagnostics is also a very big market for optical sensors [4,6]. Fortunately, MIR sensors are already finding applications in these areas. MIR sensors play a significant role in pharmaceutical testing. The already-known techniques as FTIR and ATR combined with ChGs offer an efficient and versatile solution for non-destructive pharmaceutical testing and analyzing hazardous chemicals, aqueous systems, and complex solid or liquid formulations. This method has been used for more than 25 years and is already well-developed [219]. However, with the advancement of technological possibilities, new ideas emerge. The trend for miniaturization, faster measurements, and processing of more data at the same time also applies to MIR sensors. Nevertheless, there have been demonstrations of MIR chip-scale chemical sensors, realized on the PIC technological platform of SOI. By proposing an innovative air-clad pedestal waveguide geometry, the transparency window for this material platform can be extended and reach wavelengths that match absorption regions for certain chemicals (gases or liquids), so an EWS mechanism can be implemented [220]. 

Koyama et al. presented an MIR spectroscopic system featuring a high-speed, wavelength-swept, and pulsed QCL designed for healthcare applications, including blood glucose measurement [6]. The system incorporated an ATR setup with a QCL equipped with a micro-electromechanical system (MEMS)-scanning grating, hollow optical fibers, and an InAsSb detector. By integrating multiple comb-shaped spectra with slight timing shifts, a continuous spectrum was generated. This method eliminated the need for complex calculations, enabling real-time acquisition of absorption spectra. Increasing the number of integrated spectra significantly improved the signal-to-noise ratio, allowing us to measure the absorption spectrum of a 0.1% aqueous glucose solution. Furthermore, the absorption spectra of human lips were recorded, demonstrating the potential for blood glucose estimation using a model equation derived through partial least squares regression analysis. Compared to conventional Fourier-transform infrared-based systems and other tunable QCL spectroscopic setups with large, movable gratings, this MEMS-scanning grating QCL system offered advantages in compactness and cost efficiency [6].

### 5.4. Non-Invasive Glucose Monitoring

MIR photonic techniques for non-invasive glucose monitoring leverage the unique properties of glucose molecules in the MIR region of the electromagnetic spectrum. One way to monitor glucose by MIR sensors is to use photoacoustic spectroscopy (PAS). Modulated MIR light induces thermal expansion in glucose molecules, generating acoustic waves proportional to glucose concentration. It is an innovative idea for a non-invasive way of in vivo glucose monitoring. However, it still needs to improve the penetration depth of MIR light and the signal interference from other blood components, though it looks promising [30,221]. Kitazaki et al. introduced MIR passive spectroscopic imaging as a novel approach for remote glucose measurement [32]. For the first time, spectroscopic imaging of thermal radiation from the human body successfully detected glucose-induced luminescence from a distance. Additionally, glucose emission spectra recorded from the wrist at regular intervals over 60 min demonstrated a strong correlation with blood glucose levels measured using an invasive sensor. This breakthrough technology holds promise for real-time monitoring of diabetic patients, enabling the detection of nocturnal hypoglycemia and hyperglycemia in broader populations. Furthermore, this approach could pave the way for innovations in remote biochemical sensing, allowing the detection of various substances without direct contact [32].

Nevertheless, not only PAS methods are incorporated for glucose detection. MIR quantum cascade laser spectroscopy can also be used for predicting blood glucose levels non-invasively in live human subjects, targeting glucose-specific vibrational absorption features within the “fingerprint region”. By employing a hollow-core fiber-based optical system and chemo-metric analysis using partial least squares regression, clinically accurate glucose predictions 84% of the time within a range of 80–160 mg/dL can be achieved. However, this method also meets the problem that MIR light cannot penetrate deep into tissues [222]. It is also worth mentioning that by using a tunable MIR quantum cascade laser, it is possible to develop a sensor that would aim not only to measure glucose but also lactate, and triglycerides in blood serum. A tunable QCL operating in the range of 1030–1230 cm^−1^ accesses the unique absorption features of the target analytes [223].

### 5.5. Cancer Detection and Biomarker Analysis

MIR spectroscopy is increasingly recognized for its transformative potential in biomedical applications, particularly in non-invasive and rapid cancer detection. One notable advancement is the Digistain method, a clinically oriented technique that employs an MIR imaging system for the precise chemical analysis of biopsy tissues. This method generates diffraction-limited chemical images by measuring MIR light absorption, enabling the identification of specific molecular biomarkers that differentiate cancerous tissues from healthy ones.

The Digistain system stands out for its speed and cost-efficiency, offering a streamlined alternative to traditional histopathology. Unlike conventional methods, which require intricate processes such as tissue slicing, staining, and subjective visual grading by pathologists, the Digistain technique provides reproducible and objective results. This innovative approach holds significant promise for improving diagnostic accuracy and reducing the time and expense associated with cancer detection [224,225,226].

The method mentioned above is already well-developed; however, other techniques can also be used for cancer detection. With the development of tunable QCL MIR light sources, new ideas and applications are emerging where they can be utilized. An innovative way of realizing vibrational spectroscopy for cancer detection can be realized by combining scattering-type Scanning Near-field Optical Microscopy (s-SNOM) with Quantum Cascade lasers. It allows ultra-high resolution and breaking the diffraction limit to be achieved, but its main disadvantage is that the system is very expensive and sophisticated [226].

Methods such as FTIR spectroscopy can also be used to identify biochemical changes in tissues and cells by analyzing their molecular vibrations. It may be used to detect several cancers, including those of the breast, colon, liver, and cervix. Its main advantages are that the method is reagent-free, rapid, non-invasive, and cost-effective, requiring minimal tissue samples [227]. Regarding non-invasive tools for early skin cancer detection, MIR spectroscopy also has a lot to offer. Fiber-optic evanescent wave spectroscopy (FEWS) can be used for detecting skin cancers such as melanoma, basal cell carcinoma (BCC), and squamous cell carcinoma (SCC), utilizing silver halide and bromide (AgClBr) flexible MIR optical fiber probes [160]. Nevertheless, methods employing other optical fiber materials such as chalcogenides are also being developed [228]. MIR methods surpass traditional histopathology by offering a non-invasive, objective, and real-time molecular analysis of tissues.

### 5.6. Defense and Security

MIR photonics is an essential and rapidly advancing field in security and defense, providing unparalleled capabilities in threat detection, imaging, communication, and countermeasures. Operating in the MIR wavelength range leverages the ability of IR radiation to perceive heat signatures and molecular fingerprints, making it particularly valuable in both military and homeland security applications. One of the most prominent uses of MIR photonics in defense is in IR imaging systems [229,230]. These systems allow for the detection of heat emitted by objects, which is especially useful for surveillance, reconnaissance, and target acquisition in low-visibility conditions such as nighttime operations, or through obscurants like smoke, fog, and dust [231]. Thermal IR cameras using MIR wavelengths are crucial for monitoring borders, identifying potential threats, and enhancing battlefield awareness by providing real-time thermal images [230].

In addition to imaging, MIR photonics is instrumental in chemical sensing and identification. Many toxic chemicals, explosives, and harmful gases have distinct absorption features in the MIR range, allowing sensors to identify and monitor them with high precision. This makes MIR technology highly effective for detecting chemical warfare agents, improvised explosive devices (IEDs), and environmental hazards, contributing significantly to both defense operations and counter-terrorism efforts [232,233]. Moreover, MIR photonics are used in secure communication systems [234]. The unique properties of MIR wavelengths, such as their resistance to atmospheric interference and their ability to transmit large amounts of data over optical fibers, make them highly suitable for secure, long-range communications in military networks [235,236].

VIGO Photonics has pioneered an innovative technology for manufacturing high-performance instruments designed to efficiently sense IR radiation across a wide spectrum, ranging from 2 to 16 μm [237]. These advanced detectors offer flexibility by operating at ambient temperatures or being enhanced with thermoelectric cooling systems for greater precision. Built for durability, they are engineered to perform reliably in extreme conditions, such as the intense heat of desert environments or the high-acceleration forces experienced in cutting-edge fighter jets. Tanks and military vehicles generate substantial heat, primarily in the MIR range, due to their engines and operational equipment. For such scenarios, long-wave infrared (LWIR) spectrum is recommended, especially in ground combat situations where factors like smoke or burning vehicles are prevalent. LWIR detectors excel at identifying and differentiating these heat-emitting objects from their environment. VIGO Photonics’ advanced detection technology allows for the precise, efficient targeting of moving vehicles, providing exceptional accuracy in the moments leading up to an attack [237].

**Figure 17:**
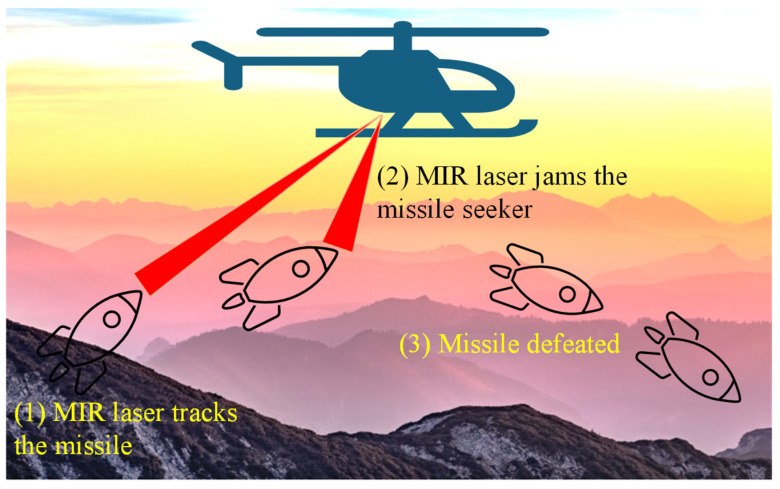
A demonstration of IRFlex’s Laser-Based IRCM technology.

Directional Infrared Countermeasures (DIRCM) and Common Infrared Countermeasures (CIRCM) are advanced anti-missile defense systems designed to protect aircraft from infrared-guided missiles [238,239]. DIRCM works by detecting incoming missile threats and then using a high-intensity laser to disrupt the missile’s IR seeker, effectively blinding it and steering it off course. CIRCM, a next-generation system, operates similarly but is more compact and modular, designed to protect a wide range of military aircraft with enhanced flexibility. Both systems rely on real-time detection and jamming, providing a critical layer of defense against heat-seeking missiles [240]. A small company based in Herndon, Virginia, focuses on developing and manufacturing fiber-optic devices for MIR applications [241]. A demonstration of IRFlex’s Laser-Based IRCM technology is presented in Figure 17. 

## 6. Challenges, Limitations, and Outlook

SOI is a strong contender for integrated photonics platforms, benefiting from its well-established technological processes derived from CMOS electronics, the extensive availability of research facilities and foundries, and its ability to integrate control electronics on the same substrate [242,243]. SOI is particularly effective for light transmission at wavelengths above 3.5 µm, though silicon oxide’s absorption beyond this range presents challenges. Addressing this issue often involves removing the silicon oxide layer beneath the waveguides, which complicates fabrication and compromises the mechanical stability of waveguides mounted on membranes or pillars [195]. Additionally, considerations such as Bragg wavelengths and lateral leakage arise when waveguides are supported by grid structures. Using submicron grids introduces further challenges in photolithography and etching, complicating wafer separation and subsequent optoelectronic packaging [244].

Even if silicon oxide’s absorption issue is resolved, silicon can transmit optical signals only up to 8 µm. Expanding the spectral range may involve germanium-based platforms. However, simple germanium waveguides on silicon substrates do not extend the spectral range due to silicon’s absorption above 8 µm. Other challenges include the significant lattice mismatch between Ge and Si layers, surface roughness of the germanium layer, high dislocation density, and low refractive index contrast between Ge and Si [245,246]. To exploit the broader spectrum of germanium beyond 8 µm, the silicon layer beneath must be removed. This solution, while effective, results in components that are mechanically fragile when mounted on membranes or gratings. Additionally, these fabrication processes are intricate and present scalability challenges [247], compounded by the limited commercial availability of germanium substrates. Alternative platforms, such as those based on chalcogenides, halides, or heavy metal oxides, also face significant hurdles [248]. These materials are often incompatible with CMOS processes, leading to limited equipment availability and underdeveloped manufacturing techniques, which increase production costs [2,20]. Chalcogenides, in particular, pose an additional concern due to their toxicity [246]. However, in 2007, a low-loss fabrication technique for single-mode chalcogenide strip waveguides that was fully compatible with Si-CMOS processing was proposed [249]. Utilizing lift-off as a novel patterning method for ChG films, this approach offered several key advantages: seamless integration with Si-CMOS workflows, the ability to produce submicron-scale single-mode waveguides, and significantly reduced sidewall roughness. High-index-contrast Ge_23_Sb_7_S_70_ strip waveguides fabricated using this method exhibited exceptional uniformity in propagation loss, achieving the lowest reported loss. Additionally, the fabrication of small-core Ge_23_Sb_7_S_70_ rib waveguides was demonstrated via lift-off, with loss values below 0.5 dB/cm [249]. 

The development of a passive integrated circuit designed for specific tasks, such as measurement, is only one step toward developing a complete sensor. For full functionality, the PIC must interface with an optical signal source, and the output signal must be detected, converted into electrical current, and further processed by electronic systems. Additionally, the entire device must be isolated from environmental influences and thermally stabilized. Each of these requirements presents distinct challenges. Monolithic integration of PICs with detector lasers on advanced silicon and germanium platforms for the MIR range remains unfeasible. While monolithic integration has been successfully achieved for group III–V material platforms [250,251], these materials are less suited for MIR photonics due to inherent limitations, preventing them from dominating this domain.

For passive platforms such as silicon and germanium, heterogeneous or hybrid integration methods, such as wafer-to-wafer or chip-to-wafer bonding, offer a solution. In this approach, passive and active components are manufactured separately—often in different production lines—before being combined. While effective, this method significantly increases the complexity of fabrication, reduces scalability, and raises production costs [112,252]. Its key advantage is the elimination of the need for active alignment during assembly [252]. Another challenge in developing miniaturized systems for MIR photonics is the requirement for specialized, efficient power supplies and control electronics to operate compact QCL lasers effectively.

Devices incorporating PICs with QCD lasers and PDs face significant challenges related to temperature stabilization. Active components, which generate substantial heat, have specific operational temperature ranges and require effective cooling mechanisms. Efficient heat dissipation and distribution across the thermoelectric module (TEM) are essential [253]. Integrating lasers into a PIC necessitates the development of structures, such as heat-conductive elements in the waveguide layer, to direct heat away from active components and into the substrate [254]. Without proper thermal management, lasers may fail to operate correctly, and passive components, like ring resonators, could become misaligned due to temperature fluctuations in the circuit.

In biosensors, waveguide surfaces can be biofunctionalized with receptors that bind to specific analytes, enabling high specificity and sensitivity [255,256]. These sensors operate at a single wavelength selected to match the analyte’s absorption characteristics. When the analyte interacts with the receptor, the evanescent field detects this binding by observing a reduction in the transmitted signal. Sensitivity can be enhanced by increasing the waveguide’s interaction area, but larger dimensions may reduce the evanescent field strength [257]. Spectroscopic sensors differ in that they do not use receptors or require surface biofunctionalization. Instead, they rely on a broadband optical source to analyze analytes near the waveguide surface through the evanescent field. These sources are particularly challenging to implement in the MIR range. The resulting spectrum often contains multiple features, requiring complex decoding that can be influenced by environmental variability [258].

A practical limitation in the development of MIR PICs is the scarcity of suitable substrates. Beyond SOI substrates, alternatives are difficult to procure, with delivery times stretching over months and inconsistent quality. This is due to the complex fabrication processes for these substrates compared to standard electronic ones and their relatively limited market demand.

Despite the larger components required for MIR wavelengths compared to visible or NIR wavelengths, PICs in this range still involve small critical dimensions. This is particularly true for sensor designs employing ring resonators, slot waveguides, or subwavelength gratings. Manufacturing such components, which have features at the scale of hundreds of nanometers, demands advanced lithographic techniques. Electron beam lithography is often used for research and prototyping due to its precision in achieving features as small as tens of nanometers without requiring expensive masks. However, its low throughput, with single processes taking hours, makes it impractical for even limited-scale production. Addressing this issue requires investment in costly wafer steppers or scanners. Additionally, improving lithographic quality necessitates using thin resist layers. While thin resists offer high pattern fidelity, they may lack the durability required for etching waveguide layers, which are typically thicker than those in CMOS technologies.

Optoelectronic packaging represents another barrier to reducing costs and improving the scalability of MIR PICs. This process is intricate, involving numerous steps that are difficult to automate. For small-scale prototyping, manual packaging is commonly used, but it significantly increases costs and limits scalability. The lack of standardized PIC parameters further complicates automation, as it hinders the packaging of circuits produced by different foundries [244].

Future advancements in MIR photonic sensors are leveraging materials like graphene and metamaterials for enhanced performance. Graphene, with its broadband optical absorption and tunable electronic properties, allows dynamic control through electrostatic gating or chemical doping, making it ideal for tunable MIR detectors and modulators [259]. Its strong plasmonic resonances in the MIR range also enhance light–matter interactions, enabling high-sensitivity PDs and modulators. However, challenges such as high optical losses and integration complexity remain. Metamaterials, with subwavelength features, offer precise control over electromagnetic waves, enabling sensitive MIR detection and functionalities like beam steering and spectral filtering. They also provide perfect absorbers for applications like thermal imaging and gas sensing. Though promising, metamaterials face challenges in scalability, optical losses, and integration. Both graphene and metamaterials hold significant potential for advancing MIR photonic sensors, with future research focused on overcoming fabrication and integration hurdles to fully exploit their capabilities [260,261].

The integration of artificial intelligence (AI) and machine learning (ML) with MIR photonic sensors holds great promise for advancing complex sample analysis, particularly in multi-component gas mixture detection [262]. AI-powered algorithms can significantly improve spectral deconvolution, enabling the precise identification of overlapping absorption features from multiple gases [263]. This is particularly beneficial for real-time environmental monitoring, industrial safety, and medical diagnostics, where accurate quantification of trace gases is critical. ML models trained on large spectral datasets can enhance sensor calibration, compensate for temperature and pressure variations, and improve sensitivity to low-concentration analytes. Additionally, AI-driven edge computing can allow for real-time, on-site data processing, reducing reliance on centralized analytical facilities and enabling autonomous decision-making. These advancements suggest that combining MIR photonic sensing with AI will lead to next-generation smart sensors capable of high-precision, real-time multi-gas analysis [1,264].

## 7. Concluding Remarks

MIR photonic sensors have emerged as powerful tools in the field of chemical sensing, due to their ability to operate in a spectral range (2–20 μm) where many organic compounds exhibit unique absorption signatures. This allows MIR sensors to detect specific chemical compositions with high sensitivity and selectivity. Key technological advances have focused on making sensors smaller, more sensitive, and better integrated with electronic systems. Progress in MIR-specific materials, such as QCLs, ICLs, and transparent materials like chalcogenides and fluoride glasses, has paved the way for more compact and cost-effective MIR sensing devices. These innovations have expanded MIR applications to areas including environmental monitoring, biomedical diagnostics, industrial process control, and defense. However, significant challenges remain, including the high cost of MIR components, limited availability of suitable materials, and issues with system integration and durability for field use.

MIR photonic sensors hold immense potential across various industries. In healthcare, for example, MIR sensors can enable rapid, non-invasive diagnostics through the detection of trace biomarkers in breath or body fluids. Environmental monitoring also stands to benefit, as MIR sensors can identify greenhouse gases and pollutants with a high degree of specificity. Similarly, MIR sensors in industrial settings enhance process control, leading to greater efficiency and precision in sectors such as petrochemical processing and food safety. Given their broad applicability and the growing demand for accurate, real-time sensing, MIR photonic sensors are well-positioned to drive advances in public health, environmental monitoring, and industrial productivity.

Future developments in MIR photonic sensors are expected to arise from continued advancements in material science, fabrication techniques, and photonic integration. Emerging materials such as graphene, metamaterials, and plasmonic waveguides show promise in further enhancing MIR sensor sensitivity. Integrated photonic circuits will also likely play a key role, allowing for smaller, lower-power, multi-functional sensor designs. As sensor technology evolves, the integration of data processing methods will likely improve real-time analysis and enable more complex applications. Together, these advancements could reshape the MIR sensor field, making it more versatile, robust, and widely applicable across diverse fields in the years to come.
